# Epigallocatechin Gallate as a Molecular Therapeutic in Heart Failure and Cardio-Oncology: Mechanistic Pathways and Translational Perspectives

**DOI:** 10.3390/ijms262110798

**Published:** 2025-11-06

**Authors:** Faika Ajaz, Jewel Haddad, Bintul Huda, Maryam Yousuf, Rajashree Patnaik, Farida Bhurka, Yajnavalka Banerjee

**Affiliations:** Department of Basic Medical Sciences, College of Medicine, Mohammed Bin Rashid University of Medicine and Health Sciences, Al Razi St, Umm Hurair 2, Dubai Healthcare City, Dubai 505055, United Arab Emiratesrajashree.patnaik@dubaihealth.ae (R.P.)

**Keywords:** epigallocatechin gallate (EGCG), catechins, green tea (*Camellia sinensis*), heart failure (HF), cardiomyopathy, myocardial infarction (MI), chemotherapy-induced cardiovascular disease, oxidative stress, Nrf2–Keap1 pathway, inflammation, fibrosis, mitochondrial dysfunction, apoptosis, autophagy, cardiac arrhythmia

## Abstract

The global burden of heart failure (HF) continues to escalate, with a lifetime risk approaching one in four adults in the United States. Concurrently, advances in cancer therapeutics have created a burgeoning population of long-term survivors, who now face the significant morbidity and mortality of chemotherapy-induced cardiovascular disease (CVD). This review addresses the critical overlap of these two pathologies, which share fundamental drivers such as oxidative stress, inflammation, and metabolic dysregulation. Epigallocatechin gallate (EGCG), the most abundant and biologically active polyphenol in green tea, has demonstrated pleiotropic bioactivity in preclinical models, encompassing potent antioxidant, anti-inflammatory, and anti-apoptotic properties. The central aim of this review is to provide a critical and comprehensive synthesis of the evidence supporting EGCG’s dual protective role. This review dissects its molecular mechanisms in modulating key pathways in HF and cardio-oncology, evaluates its translational potential, and importantly, delineates the significant gaps that must be addressed for its clinical application. This analysis uniquely positions EGCG not merely as a nutraceutical, but as a multi-target molecular therapeutic capable of simultaneously addressing the convergent pathological cascades of heart failure and cancer-related cardiotoxicity. The synthesis of preclinical evidence with a critical analysis of its translational barriers offers a novel perspective and a strategic roadmap for future research.

## 1. Introduction

Heart failure (HF) remains a major public health crisis with a profound impact on global morbidity, mortality, and economic stability [1]. In the United States, approximately 6.7 million adults over the age of 20 live with heart failure, a figure projected to rise to 8.7 million by 2030, 10.3 million by 2040, and a staggering 11.4 million by 2050 [2]. The lifetime risk of developing HF has increased to 24%, meaning that approximately one in four individuals will experience this condition in their lifetime [3]. A concerning demographic shift is underway, with a disproportionate increase in the prevalence and mortality of HF observed among younger adults (35–64 years) and certain racial and ethnic groups, particularly Black and Hispanic individuals, who demonstrate higher incidence and mortality rates compared to other populations [4]. This rising disease burden is compounded by a dramatic economic cost; annual cardiovascular healthcare costs are projected to almost quadruple between 2020 and 2050 [5].

Concurrently, the therapeutic landscape of HF has evolved considerably. The introduction of sodium–glucose cotransporter-2 (SGLT2) inhibitors has provided significant improvements in morbidity and mortality across HF subtypes, redefining standard of care [6]. These agents have been shown to reduce clinical events with early and sustained benefits regardless of ejection fraction, diabetic status, or care setting. Originally developed for type 2 diabetes, SGLT2 inhibitors (also known as “flosins”) have revolutionized HF management and are now considered a core component of modern foundational therapy. Their primary mechanism involves reducing glucose and sodium reabsorption in the proximal renal tubule, leading to glycosuria, osmotic diuresis, and favorable hemodynamic effects. Beyond these actions, they exert pleiotropic effects, including modulation of oxidative stress, inflammation, and endothelial function. Large multicenter trials consistently demonstrate reductions in HF hospitalizations across the spectrum of systolic function, underscoring their broad clinical applicability. Clinicians should therefore be familiar with their use and potential adverse effects to optimize patient outcomes.

Similarly, novel nonsteroidal mineralocorticoid receptor antagonists such as finerenone have shown promise in improving both cardiovascular and renal outcomes, further broadening treatment strategies [7]. A recent meta-analysis encompassing more than 19,000 patients from pivotal RCTs (FIDELIO-DKD, FIGARO-DKD, and FINEARTS-HF) demonstrated a 20% reduction in HF hospitalization risk (HR 0.80, 95% CI: 0.72–0.90) and a 14% reduction in all-cause mortality (RR 0.86, 95% CI: 0.77–0.97) with finerenone, though effects on cardiovascular death and renal failure require further clarification. Hyperkalemia remains the most notable adverse effect. These findings highlight finerenone’s potential role in reshaping long-term HF and CKD management, complementing established therapies.

At the same time, the field of cardio-oncology has emerged as a vital discipline, addressing the cardiovascular consequences of cancer and its treatments [8]. As cancer survival rates have improved over the past several decades, the long-term cardiotoxic effects of life-saving therapies, such as anthracyclines and targeted agents, are increasingly recognized as a major contributor to long-term morbidity and mortality in cancer survivors [9].

Chemotherapy-related cardiac dysfunction is increasingly recognized, with an overall pooled incidence of 63.21 per 1000 person-years [10]. The risk varies by agent and regimen: anthracyclines may cause cardiotoxicity in up to 48% of patients at higher cumulative doses [11], while trastuzumab, especially when combined with anthracyclines, carries rates of up to 28% [12]. Against the backdrop of rising heart failure in younger populations and a growing cohort of cancer survivors, these treatment-induced complications highlight an urgent need for targeted preventive and therapeutic strategies [13,14].

At a fundamental level, the pathophysiology of chronic heart failure and cancer is not entirely distinct but is driven by a shared set of cellular and molecular alterations [15]. These shared drivers include persistent oxidative stress, chronic systemic and localized inflammation, mitochondrial dysfunction, and metabolic reprogramming [15,16,17]. For example, cancer patients frequently experience systemic effects such as oxidative stress and inflammation, which can contribute to the development of traditional cardiovascular risk factors such as hypertension and dyslipidaemia [18]. Cancer and its treatment modalities have long been recognized to induce CV functional decline in individuals with cancer, encompassing conditions such as left ventricular diastolic dysfunction, thromboembolic diseases, and HTN [19]. The notion that these two seemingly disparate diseases share core pathological pathways provides a strong rationale for exploring multi-target therapeutic agents that can simultaneously intervene in both disease states [15].

Epigallocatechin gallate (EGCG), a natural polyphenol found in high concentrations in green tea, has attracted significant scientific interest due to its remarkable ability to modulate multiple intracellular signaling cascades [20,21]. This multi-target activity is highly advantageous in complex, multifactorial diseases like HF and cardio-oncology, contrasting with the single-target approach of many conventional drugs [22,23]. This review will critically examine the evidence for EGCG’s intervention in these shared pathways, aiming to bridge the gap between its known nutraceutical benefits and its potential as a pharmacological agent.

This review is structured to guide the reader from the foundational principles of EGCG to its clinical and translational future. The initial sections outline the literature search strategy and summarize EGCG’s key biochemical properties. Subsequent sections will be dedicated to a detailed, mechanistic analysis of its effects on heart failure and cardio-oncology. The final sections will provide a rigorous translational perspective, outlining current limitations and a strategic roadmap for future research.

## 2. Methodology

The systematic literature search was conducted across multiple scientific databases, including PubMed, Scopus, Web of Science, and Embase. A comprehensive list of keywords was used, including “EGCG,” “green tea catechins,” “heart failure,” “cardiomyopathy,” “myocardial infarction,” “cardio-oncology,” “cardiotoxicity,” “doxorubicin,” “trastuzumab,” and “tyrosine ดรพำkinase inhibitors.” The search was limited to English language, peer-reviewed articles published between the years 2000 and 2025. The inclusion criteria specified the selection of both preclinical (in vitro and animal) and clinical studies. Exclusion criteria included non-peer-reviewed literature and studies focused on other nutraceuticals. The screening process involved an initial review of titles, followed by abstract screening, and finally, a full-text review to ensure relevance and quality. The literature selection process will be visually represented in a PRISMA-style flow diagram to ensure the transparency and reproducibility of the review’s methodology.

Animated figures presented in this manuscript are original creations developed by the authors using https://www.BioRender.com (accessed on 1 September 2025), version 2024.3, adhering to institutional licensing agreements and figure preparation guidelines, similar to the approach used in a review by Al-Kabani et al. [24]. None of the figures presented in this manuscript are copyrighted or have been replicated from external sources.

## 3. Chemical and Molecular Structure of Epigallocatechin Gallate

**Figure 1:**
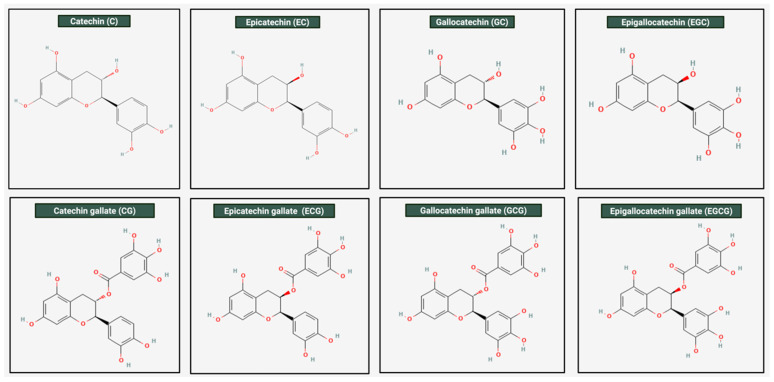
Molecular structures of catechin found in green tea. This figure illustrates the chemical structures of the different types of catechins found in green tea. As shown, all of them contain the core flavan-3-ol structure; however, variations are present. Among them, EGCG is the largest and most complex of the catechins. It has a gallate group attached to the hydroxyl group on the C-ring, an extra hydroxyl group on the B-ring, making it a “tri-hydroxy” structure instead of a di-hydroxy structure. These molecular properties, particularly the gallate group, contribute to its relatively net hydrophobic nature. EGCG is the most abundant catechin found in green tea.

Catechins are biologically active compounds naturally found in green tea leaves (*Camellia sinensis*). Among them, one of the most abundant and reactive compounds is Epigallocatechin gallate (EGCG) [25]. The IUPAC name EGCG is [(2R,3R)-5,7-dihydroxy-2-(3,4,5-trihydroxyphenyl)-3,4-dihydro-2H-chromen-3-yl] 3,4,5-trihydroxybenzoate with a molecular weight of 458.4 g/mol. EGCG is soluble in solvents such as water, ethanol, methanol, acetone, tetrahydrofuran, and pyridine [20]. Figure 1 illustrates the different molecular structures of catechins, highlighting the structural variation among these bioactive compounds.

## 4. Strategies for Isolation of Epigallocatechin Gallate from Green Tea

High performance liquid chromatography (HPLC): High-performance liquid chromatography (HPLC) remains the most widely applied technique for quantifying EGCG, with reversed-phase HPLC offering high sensitivity and reproducibility in separating catechins from complex matrices [26]. Recent methodological refinements, including quality-by-design approaches such as Taguchi orthogonal array design, have further improved detection limits and recovery, supporting its robustness for quantitative and translational applications [27].

One of the key advantages of HPLC is that it allows the simultaneous determination of multiple compounds with good separation performance, and its compatibility with a variety of detectors adds flexibility for different analytical needs [27,28]. However, HPLC does have limitations. Firstly, it can be time consuming and less suitable for high-throughput analysis due to long run times. In addition, HPLC presents challenges such as high operational costs, the requirement for specialized expertise, and susceptibility to contamination, which may affect reproducibility [29]. Advances such as ultra-high-performance liquid chromatography (UHPLC) have helped overcome some of these issues by enabling faster separations (5–10 times quicker) with improved resolution compared to conventional HPLC [28].

Thin-Layer Chromatography (TLC): TLC is a technique that relies on the fact that different catechins interact differently with the stationary layer and the solvent system due to their differing affinities, allowing their separation according to their molecular characteristics [30]. This method is simple and fast at separating the specific substances from their complex samples [31]. This is why TLC is widely used for the extraction of EGCG. Both conventional TLC and its advanced form, high-performance TLC (HPTLC), are considered versatile techniques capable of handling multiple samples in parallel with relatively high throughput [32]. The method is valued because it is inexpensive, simple to perform, and requires minimal instrumentation. Development times are short, and even with its straightforward setup, the technique can offer considerable sensitivity and reliable reproducibility [33]. Furthermore, the thin-layer format is advantageous when dealing with complex matrices, since it accommodates higher sample loads and allows flexible visualization and detection. TLC has key limitations as well. The method is strongly influenced by environmental factors such as humidity and the preconditioning of plates, which can introduce variability and errors [34]. Compared with HPLC, it generally provides lower resolution and poorer quantitative accuracy, restricting its use when precise separation or exact concentration measurements are required [30].

Supercritical fluid extraction (SFE): SFE commonly employs carbon dioxide as the solvent. When CO_2_ is pressurized above its critical point, it takes on both gas-like and liquid-like properties, allowing it to penetrate compounds efficiently and dissolve target compounds such as catechins particularly EGCG [35]. A major advantage of SFE is that it operates at relatively mild temperatures, which helps protect compounds from heat-related breakdown and preserves their biological activity [36]. The process also has short extraction times, can be carried out with little or no use of organic solvents, and causes minimal degradation [37]. SFE has practical drawbacks as well. The method requires high-pressure equipment and specialized infrastructure, which come with substantial upfront costs. These expenses make large-scale implementation in industry expensive compared to more conventional extraction techniques [36].

In summary, among the available strategies, HPLC remains the gold standard for the isolation and quantification of EGCG because of its superior resolution, reproducibility, and compatibility with multi-detector platforms, making it highly adaptable for both research and industrial settings. Nevertheless, the operational complexity and cost associated with HPLC highlight the need for ongoing methodological innovation. Future research should focus on refining UHPLC and HPTLC platforms to balance speed, throughput, and quantitative accuracy, while also exploring hybrid approaches such as coupling chromatography with mass spectrometry or capillary electrophoresis for deeper metabolite profiling. In parallel, greener and more sustainable extraction technologies, such as supercritical fluid chromatography, pressurized liquid extraction, and membrane-assisted separation, should be systematically evaluated to reduce solvent usage and improve scalability. Looking forward, the integration of miniaturized microfluidic chromatographic devices and AI-driven optimization of separation parameters represents a promising frontier, potentially enabling rapid, high-precision purification of EGCG with reduced environmental and economic burden.

## 5. Geographical Variations in Green Tea Species and Epigallocatechin Gallate Content

Beyond the choice of purification method, careful consideration of the inherent EGCG content across different green tea sources is equally critical, as it directly influences dosing strategies, safety margins, and the consistency of therapeutic outcomes. Table 1 shows the different types of green tea species and their respective EGCG content. Conceptualizing the EGCG content in different types of green tea leaves is crucial for assessing its potential use as a therapeutic agent, and serves as a metric for standardizing dosage, which is essential for achieving a consistent and reproducible clinical effect. Different green tea cultivars and processing methods can lead to significant inter-variation. Furthermore, accessibility also plays a role. While Korean Green Tea (Woojeon) with 105.37 mg/g has the highest EGCG content, it is less accessible compared to green tea species such as Matcha. Yet, even within Matcha there are also multiple grades (average, ceremonial, and culinary) depending on cultivation, thus carrying their own distinct EGCG content.

Moreover, storage conditions have a significant impact on the stability of EGCG. It readily undergoes oxidation and epimerization to gallocatechin gallate (GCG), processes that are accelerated by elevated temperature, neutral to alkaline pH, oxygen exposure, and light. As a result, the content of EGCG in green tea infusions or extracts stored in under-suboptimal conditions gradually decreases. Commercially prepared beverages, for example, often contain significantly lower levels of EGCG than freshly brewed tea, largely due to degradation during manufacturing and storage. Optimal preservation requires acidic environments (pH ~3–4), low temperatures (4 °C), exclusion of oxygen, and protection from light, conditions that significantly slow down degradation. These findings highlight the need to consider storage variables in both experimental designs and translational applications, as the apparent concentration and biological activity of EGCG may be underestimated if stability is not rigorously controlled [38,39,40].

Beyond issues of storage stability, the absolute EGCG content in tea leaves is equally critical, as it determines the effective therapeutic dose and ensures that potential toxicity thresholds are not exceeded. Ramachandran et al. demonstrated the effect of pure EGCG repeated doses in mice, observing dose-dependent hepatotoxicity [41].Thus, evaluation of safe EGCG concentration is vital to ensure a more patient-complaint safety profile. Lastly, population variability plays a significant role in EGCG activity. When studying the effect of extrinsic factors, such as genetics, sex, age, patient-specific factors, knowing and standardizing the EGCG content from its source is essential to ensure robust and reliable data.

**Table 1:** Variation in EGCG content and bioaccessible dose among commonly consumed green teas.

Green Tea Type	Geographical Area of Origin	Processing Method/Cultivar	EGCG Content in Dry Leaf (mg/g)	EGCG per Serving (mg) (Based on 2 g Dry Tea or Powder)	Serving Volume (mL)	EGCG Concentration per Serving (µM)	Reference	Remarks on EGCG Measurement and Brewing
General Green Tea (Infusion)	Global	Various	N/A	165	240	1499.0	[42]	General average for brewed green tea.
General Green Tea (Infusion)	Global	Various	N/A	90	200	981.7	[43]	Based on 2.5 g tea leaves per 200 mL water.
General Green Tea (Dry Leaves)	Global	Various	73.8	147.6	N/A	N/A	[42]	EGCG content in dried leaves, not brewed.
Matcha	Japan	Powdered, Shade-grown	50.5–56.6 (avg. ceremonial: 56.6; culinary: 50.5)	101–113	100	~22–24	[44]	Based on 2 g powder per cup. Consuming whole leaf.
Gyokuro	Japan	Shade-grown	53.31	106.62	240	969.4	[45]	EGCG in dry leaf.
Gyokuro (Infusion)	Japan	Shade-grown	N/A	268.09	240	2437.0	[45]	Infusion from 10 g leaves in 60 mL water at 60 °C for 2 min. Highly concentrated.
Sencha (Superior)	Japan	Sun-grown	67.45	134.90	240	1226.2	[45]	EGCG in dry leaf.
Sencha (Superior Infusion)	Japan	Sun-grown	N/A	74.41	240	676.0	[45]	Infusion from 6 g leaves in 170 mL water at 70 °C for 1 min.
Sencha (Standard)	Japan	Sun-grown	62.16	124.32	240	1131.0	[45]	EGCG in dry leaf.
Sencha (Standard Infusion)	Japan	Sun-grown	N/A	91.30	240	830.0	[45]	Infusion from 6 g leaves in 260 mL water at 90 °C for 1 min.
Sencha (Deep-Steamed)	Japan	Sun-grown, Deep-steamed	63.51	127.02	240	1155.0	[45]	EGCG in dry leaf.
Sencha (Deep-Steamed Infusion)	Japan	Sun-grown, Deep-steamed	N/A	114.20	240	1038.0	[45]	Infusion from 6 g leaves in 260 mL water at 90 °C for 1 min.
Sencha (Infusion)	Japan	Sun-grown	N/A	124 mg/100 mL	100	2705.4	[46]	EGCG content per 100 mL of infusion.
Tamaryokucha (Pan-Fired)	Japan	Pan-fired	64.29	128.58	240	1168.0	[45]	EGCG in dry leaf.
Tamaryokucha (Pan-Fired Infusion)	Japan	Pan-fired	N/A	65.72	240	597.1	[45]	Infusion from 6 g leaves in 260 mL water at 90 °C for 1 min.
Tamaryokucha (Steamed)	Japan	Steamed	61.61	123.22	240	1120.0	[45]	EGCG in dry leaf.
Tamaryokucha (Steamed Infusion)	Japan	Steamed	N/A	90.00	240	818.0	[45]	Infusion from 6 g leaves in 260 mL water at 90 °C for 1 min.
Lu’an Guapian (HSGP)	Anhui, China (Huoshan County)	Traditional Processing	110.69	221.38	240	2013.7	[47]	EGCG in dry leaf (WT%).
Lu’an Guapian (JZGP)	Anhui, China (Jinzhai County)	Traditional Processing	87.18	174.36	240	1585.5	[47]	EGCG in dry leaf (WT%).
Lu’an Guapian (YAGP)	Anhui, China (Yu’an District)	Traditional Processing	80.23	160.46	240	1459.7	[47]	EGCG in dry leaf (WT%).
Lu’an Guapian (IMGP)	Anhui, China (Inner Mountain)	Traditional Processing	92.75	185.50	240	1687.2	[47]	EGCG in dry leaf (WT%).
Lu’an Guapian (OMGP)	Anhui, China (Outer Mountain)	Traditional Processing	79.33	158.66	240	1443.5	[47]	EGCG in dry leaf (WT%).
Korean Green Tea (Woojeon)	Korea	Early Plucking Period	105.37	210.74	240	1916.6	[48]	EGCG in dry leaf.
Korean Green Tea (Sejak)	Korea	Mid Plucking Period	103.95	207.90	240	1890.3	[48]	EGCG in dry leaf.
Korean Green Tea (Joongjak)	Korea	Late Plucking Period	111.59	223.18	240	2030.0	[48]	EGCG in dry leaf.
Korean Green Tea (Daejak)	Korea	Latest Plucking Period	112.86	225.72	240	2053.3	[48]	EGCG in dry leaf.
Jeju Green Tea (Steamed)	Jeju, Korea	Steaming	24.0 (Extract)	48.0 (Extracted)	240	436.0	[49]	EGCG extracted from dry tea into infusion (mg/g dry tea). Assumed 2 g dry tea serving.
Jeju Green Tea (Pan-fired)	Jeju, Korea	Pan-firing	31.8 (Extract)	63.6 (Extracted)	240	578.0	[49]	EGCG extracted from dry tea into infusion (mg/g dry tea). Assumed 2 g dry tea serving.
Jeju Green Tea (Steamed and Pan-fired, Light)	Jeju, Korea	Steaming and Pan-firing	20.2 (Extract)	40.4 (Extracted)	240	367.0	[49]	EGCG extracted from dry tea into infusion (mg/g dry tea). Assumed 2 g dry tea serving.
Jeju Green Tea (Steamed and Pan-fired, Heavy Roast)	Jeju, Korea	Steaming and Pan-firing, Heavy Roasting	42.3 (Extract)	84.6 (Extracted)	240	769.0	[49]	EGCG extracted from dry tea into infusion (mg/g dry tea). Assumed 2 g dry tea serving.
Boseong Green Tea (Pan-fired)	Boseong, Korea	Pan-firing	39.9 (Extract)	79.8 (Extracted)	240	725.0	[49]	EGCG extracted from dry tea into infusion (mg/g dry tea). Assumed 2 g dry tea serving.
Hangjou Green Tea (Pan-fired)	Hangjou, China	Pan-firing	36.9 (Extract)	73.8 (Extracted)	240	671.0	[49]	EGCG extracted from dry tea into infusion (mg/g dry tea). Assumed 2 g dry tea serving.
Shizuoka Green Tea (Steamed)	Shizuoka, Japan	Steaming	24.5 (Extract)	49.0 (Extracted)	240	445.0	[49]	EGCG extracted from dry tea into infusion (mg/g dry tea). Assumed 2 g dry tea serving.
Longjing (Dragon Well)	Hangzhou, Zhejiang, China	Pan-fired	Variable	Variable	240	Variable	[50]	Known for high thiamine; EGCG present but specific values per serving not consistently provided.
Biluochun (Green Snail Spring)	Jiangsu, China	Traditional Processing	Variable	Variable	240	Variable	[50]	Rich in polyphenols, high antioxidant level. Specific EGCG per serving not provided.
Huangshan Maofeng	Anhui, China	Traditional Processing	Variable	Variable	240	Variable	[50]	Rich in antioxidants, including EGCG, but specific values per serving not provided.

## 6. Determinants of Epigallocatechin Gallate Bioavailability and Absorption

### 6.1. Metabolic Barriers to Epigallocatechin Gallate Bioavailability

**Figure 2:**
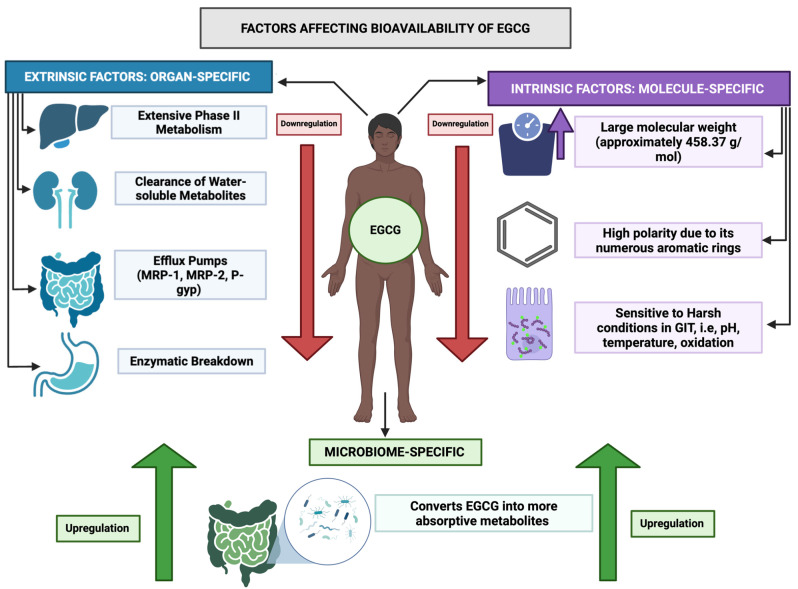
Overview of factors affecting bioavailability of EGCG. This figure highlights the different factors affecting the absorption, accumulation, and bioavailability of EGCG from the human GIT to target cells. There are certain factors that correspond to the EGCG molecule itself—molecular weight, polarity, and chemical instability; these aspects make it less ideal of a molecule to be passively absorbed along the size-selective, lipophilic phosphodiester bilayer of the enterocytes. In addition, factors such as liver’s first pass metabolism, fast clearance by the renal system, efflux mechanism in the GIT, and enzymatic breakdown in the gut further reduce EGCG’s bioavailability in the human tissues. However, the gut microbiome is one factor present in the human colon that allows EGCG to be absorbed more readily. Via microbiome-mediated enzymatic processes, it allows EGCG to be molecularly changed to facilitate its passive diffusion across the human gut.

EGCG demonstrates poor oral bioavailability, largely due to rapid clearance and extensive phase II metabolism occurring primarily in the intestine and liver [20]. Figure 2 highlights the different factors affecting the absorption, accumulation, and bioavailability of EGCG.

After absorption, EGCG undergoes methylation, sulfation, and glucuronidation, processes which markedly reduce the concentration of free, bioactive EGCG available in systemic circulation [25]. These conjugated metabolites are generally more water-soluble and readily excreted, which further decreases overall bioavailability. Importantly, metabolic conjugation lowers systemic exposure to the parent EGCG compound and alters biological activity; although some conjugates retain partial antioxidant properties, they are usually less potent than free EGCG. From a therapeutic standpoint, this extensive metabolism is not ideal, as the most robust bioactivity appears to come from unmetabolized EGCG [25]. Therefore, to optimize its translational potential, strategies to enhance the stability of free EGCG, such as through nano-formulations or chemical modifications, are warranted.

### 6.2. Microbiome-Associated Epigallocatechin Gallate Pharmacokinetics and Absorption

The gut microbiome has a major pharmacokinetic significance in EGCG metabolism in the human gut. Table 2 highlights the representative microbiota involved in metabolic processing of EGCG in the body along with their biological relevance.

Only a small fraction of dietary polyphenols is absorbed in the small intestine; most bypass it and reach the colon, where the microbial community plays a central role in their metabolism [56]. The microbiome-driven transformations of EGCG occur through the following five major processes: degalloylation, ring fissure, dehydrogenation, hydroxylation, and carboxylation, each of which critically shapes its pharmacokinetic journey. Using ultrahigh performance liquid chromatography coupled with hybrid quadrupole Orbitrap mass spectrometry (UHPLC-Q-Orbitrap-MS), Liu et al. demonstrated that EGCG undergoes sequential microbial degradation, including ester hydrolysis, C-ring opening, A-ring fission, dehydroxylation, and aliphatic chain shortening, ultimately yielding smaller, bioactive metabolites [51].

One of the key steps is degalloylation, mediated by esterases of *Bifidobacterium* and *Lactobacillus*, which produces epigallocatechin (EGC) and gallic acid (GA). This process not only facilitates the absorption of EGC but also liberates GA locally, where it exerts anti-inflammatory and antioxidant effects [57]. GA has shown cardioprotective benefits in multiple models; Jin et al. demonstrated improved outcomes in a pressure overload-induced HF mouse model and rat cardiac fibroblasts [58], while Badavi et al. reported enhanced activity of scavenging enzymes such as lactate dehydrogenase, superoxide dismutase, catalase, and glutathione peroxidase in a Wistar rat myocardial infarction (MI) model [59]. These findings were corroborated by Priscilla et al., who showed that GA reduced oxidative stress in isoproterenol-induced MI [60]. Thus, microbial production of GA from EGCG illustrates how beneficial effects can arise even before the parent compound reaches target tissues.

EGC itself, though less potent than EGCG, also contributes to antioxidant defense. He et al. showed that catechins, including EGC, mitigated H_2_O_2_-induced cell damage in HT22 cells, improving viability and reducing ROS levels, as confirmed by flow cytometry [61]. The presence of a pyrogallol group on the C-ring of EGC underlies its ability to scavenge ROS [62]. Together, these data suggest that both EGCG and its microbial metabolites extend protective antioxidant functions relevant to HF.

Other transformations, including dehydrogenation and reduction in the A- and B-rings, generate phenylpropionic and phenylacetic acids [51]. These smaller molecules diffuse readily across enterocyte membranes, increasing systemic absorption. Importantly, phenylpropionic acid also exhibits antioxidant activity, as confirmed by visible spectroscopy, which shows that hydroxyl substitutions on the aromatic ring enhance its radical-scavenging capacity [63]. Ultimately, EGCG metabolism yields end-products that are excreted in urine through the combined activity of mixed microbiota, which may serve as useful biomarkers of microbiota-driven pharmacokinetics [20,64].

The breakdown from the parent compound EGCG to its smaller active metabolites does influence its bioavailability and cellular activity, hence the microbiome profile and inter-individual differences amount populations which may significantly influence EGCG uptake metabolite-formation. Researchers refer to these as “gut metabotypes” op-r “gut microbiota-associated metabotypes” [65].

Case in point—Liu et al. observed these so-called gut metabotypes. Differences in metabolite profiles—such as gallic acid, pyrogallol, phenylpropane-2-ols, and phenyl-γ-valerolactones—were directly correlated with the abundance of specific microbial taxa identified via 16S rRNA sequencing [66]. Importantly, gut dysbiosis is linked to clinical conditions including cancer and cardiovascular disease, which may further change the metabolic fate of EGCG. This interaction emphasizes the necessity of considering the microbiota as a potential modulator of EGCG’s therapeutic effects in addition to its role as a metabolic barrier. Therefore, to assess EGCG pharmacokinetic and absorption and its further development as a therapeutic agent, gut metabotypes must be considered.

In summary, EGCG escapes small-intestinal absorption and undergoes microbiota-driven biotransformations (degalloylation, ring/C-ring fission, dehydroxylation, reduction, chain shortening) that generate bioactive metabolites (e.g., EGC, GA, phenylpropionic/phenylacetic acids, γ-valerolactones). Because these metabolites materially contribute to systemic exposure and effect, inter-individual “gut metabotypes” and dysbiosis become key determinants of pharmacokinetic and pharmacodynamic variability.

Considering the evidence presented, future work should advance from a compound-centric approach toward a systems pharmacokinetic/pharmacodynamic (PK/PD) framework, integrating metagenomics, targeted and untargeted metabolomics, and lipidomics with physiologically based pharmacokinetic (PBPK) models of the gut–liver axis. Stable-isotope–resolved metabolomics (SIRM) [67], employing uniformly 13C-labeled EGCG and 13C-gallic acid could be particularly valuable in resolving metabolite origin and carbon flux into GA, pyrogallol, phenyl-γ-valerolactones, phenylpropionates, and hippurate. Such studies should be coupled with high-resolution UHPLC-HRMS/MS workflows [68], using parallel reaction monitoring (PRM) [69] and data-independent acquisition (DIA) [70], to enable absolute quantitation of EGCG conjugates generated through UGT, SULT, and COMT activity, as well as transporter-dependent excretion mediated by ABCG2/BCRP, ABCB1/P-gp, and ABCC2/MRP2. Another critical area will be the definition of microbiota-associated metabotypes, using unsupervised clustering of metabolite ratio features (for example, Σvalerolactones/GA, GA-sulfate/GA-glucuronide, or valerolactone-glucuronides/valerolactone-sulfates), which can be mechanistically linked to taxa-level enzyme capacities such as esterases, reductases, and dehydroxylases. These associations should then be validated by employing anaerobic gnotobiotic consortia and CRISPR-edited strains of relevant bacteria, including Flavonifractor, Eggerthella, and Bifidobacterium [71,72,73]. Parallel efforts must also address the contribution of host lipidomics [74], with both shotgun and targeted approaches required to quantify how bile-salt pools, mixed micelles, and phospholipid composition [75] determine luminal solubilization and enterocyte uptake of EGCG and its metabolites [76]. Likewise, profiling of microbial-derived short-chain fatty acids (SCFAs) and secondary bile acids using LC-MS/MS and GC-MS [77] will provide insight into their role as regulators of transporter expression (e.g., OATP1A2/2B1, SLC22 family, MCTs), and efflux systems (BCRP, P-gp, MRPs) [78] as well as their influence on intestinal barrier integrity [79].

To experimentally capture these dynamics under physiologically relevant conditions, microphysiological systems should be prioritized. Anaerobic gut-on-chip models co-cultured with defined microbial consortia, when coupled with liver-on-chip platforms [80], offer the potential to parameterize intestinal and hepatic clearance, as well as conjugation kinetics, under authentic bile-acid and lipid milieus. Such systems can be complemented by MALDI mass spectrometry imaging (MALDI-MSI) to map the tissue distribution of EGCG versus gallic acid and related metabolites.

From a translational standpoint, the systematic evaluation of bioenhancer interactions represents another important research axis. For example, screening piperine, a piperidine alkaloid, and related structural scaffolds against UGT1A1/1A9, SULT1A1, COMT, and efflux transporters such as BCRP and P-gp using vesicle systems and Caco-2/MDCK transporter assays will help to establish reliable inhibition constants (Ki/IC50). These values should be integrated into PBPK models and drug–drug interaction (DDI) risk assessments, with subsequent validation in microbially stratified crossover trials to confirm clinical relevance.

Regional population specificity must also be addressed, particularly in Gulf-region cohorts, where dietary patterns, baseline bile-acid/lipidome composition, antibiotic exposure, and microbiome diversity [81], are likely to shape EGCG metabolism. By jointly modeling these factors, researchers can establish metabotype-aware reference intervals and dosing algorithms tailored to specific populations. In parallel, pre-analytical variables, including brewing conditions, storage pH, oxygen and light exposure, and temperature should be rigorously standardized. Reporting metabolite panels, including urinary hippurate and phenyl-γ-valeric acids, as pharmacokinetic endpoints rather than focusing exclusively on parent EGCG, would provide a more accurate reflection of systemic exposure.

Finally, advances in artificial intelligence and machine learning offer transformative potential. Multi-view variational models and graph-based networks can integrate multi-omic datasets—including metagenome, metabolome, lipidome, and transporter genotypes—to generate predictive exposure–response maps at the individual level [82]. This strategy could ultimately enable precision nutraceutical deployment of EGCG, where dosing and formulation are informed by a subject’s microbiota composition, lipidome profile, and genetic determinants of transport and metabolism.

## 7. Polypharmacy and Epigallocatechin Gallate–Drug Interactions

In clinical practice, polypharmacy introduces another critical variable that influences EGCG bioavailability. Table 3 illustrates the multiple drug class, their representative drugs, and consequence of EGCG bioavailability. EGCG and green tea catechins are recognized inhibitors of intestinal uptake transporters (notably OATP1A2/2B1) and modulators of efflux pumps such as P-glycoprotein (P-gp) [83]. These interactions can significantly reduce systemic exposure to co-administered drugs. For example, EGCG markedly decreases plasma concentrations of the β-blocker nadolol [84,85] and the ACE inhibitor lisinopril [86], both standard therapies in HF management, by impairing intestinal uptake. Similar transporter-mediated reductions have been documented with fexofenadine, an OATP1A2 substrate [87]. In oncology, EGCG has a particularly concerning interaction with the proteasome inhibitor bortezomib, where direct chemical antagonism abolishes its cytotoxic efficacy [88].

Given these clinically relevant interactions, future research must prioritize systematic transporter–substrate mapping of EGCG and its metabolites using high-throughput in vitro assays, integrated PBPK/DDI modeling, and stratified clinical validation, to establish evidence-based guidelines for co-administration in polypharmacy settings. Case in point, in heart failure, impaired uptake of β-blockers such as nadolol or ACE inhibitors like lisinopril could compromise hemodynamic stability [86,89], while in oncology, direct antagonism of bortezomib [90,91] underscores the potential for EGCG to undermine chemotherapeutic efficacy. Addressing such scenarios requires not only mechanistic dissection of transporter and metabolic pathways but also carefully designed clinical trials that evaluate EGCG use in patients receiving cardiovascular and anticancer therapies.

**Table 3:** Drugs affecting EGCG bioavailability.

Drug Class	Representative Drugs	Indication	Mechanism of Interaction	Effect on EGCG Bioavailability	Notes/Clinical Relevance	Refs.
COMT (Catechol-O-methyltransferase) Inhibitors	Entacapone, Tolcapone	Parkinson’s disease	Inhibit methylation of EGCG	↑ Plasma EGCG levels, prolonged half life	Risk of higher systemic EGCG exposure; may increase adverse effects (e.g., hepatotoxicity)	[92]
UGT (UDP-glucuronosyltransferase) Inhibitors	Valproic acid	Epilepsy	Block EGCG glucuronidation in intestine/liver	↑ Bioavailability	Drug–nutrient competition; caution with chronic use	[93,94]
	Probenecid	Gout				
SULT (Sulfotransferase) Inhibitors	Diclofenac, Celecoxib	Inflammatory conditions, pain, arthritis	Reduce sulfation of EGCG	↑ Systemic exposure	Possible synergy in inflammation models	[95]
P-gp (P-glycoprotein) Inhibitors	Verapamil	Hypertension, arrhythmias	Prevent EGCG efflux from intestinal cells	↑ Absorption and plasma concentrations	High risk for pharmacokinetic interactions with narrow therapeutic index drugs	[96,97,98]
	Cyclosporine	Immunosuppression				
	Quinidine	Arrythmias				
OATP (Organic Anion Transporting Polypeptide) Substrates/Inhibitors	Statins	Hyperlipidemia, cardiovascular risk reduction	Competition on intestinal uptake transporters	↓ Oral uptake of EGCG OR altered statin pharmacokinetics	EGCG may reduce statin absorption (bidirectional effect)	[99,100]
	Rifampicin	Tuberculosis				
Proton Pump Inhibitors (PPIs)	Omeprazole, Pantoprazole	GERD, peptic ulcers	Increase gastric pH → reduce EGCG degradation	Potential ↑ stability but ↓ solubility depending on formulation	Effect varies with formulation (capsules vs. tea extract)	[101]
Antibiotics (Broad Spectrum)	Ciprofloxacin, Amoxicillin–Clavulanate	Broad-spectrum bacterial infection	Disrupt gut microbiota metabolism of EGCG	↓ Formation of valerolactone/phenolic metabolites; altered bioactivity	Reduces health benefits mediated by microbiota-derived metabolites	[102]
Iron Supplements	Ferrous sulfate, Ferric chloride	Iron deficiency anemia	Chelates EGCG, forming insoluble complexes	↓ EGCG absorption	Should avoid co-administration of EGCG-rich tea with iron	[103]
Anticoagulants/Antiplatelets	Warfarin	Thrombosis prevention and treatment, atrial fibrillation	Not directly altering PK but EGCG itself has antiplatelet/anticoagulant properties	↑ Bleeding risk despite bioavailability changes	Clinical safety concern	[104,105,106,107]
	Aspirin, Clopidogrel	Cardiovascular disease prevention, antiplatelet therapy				
Protease Inhibitors (HIV Drugs)	Ritonavir, Saquinavir	HIV infections	EGCG can inhibit CYP3A4 and P-gp, altering drug PK; reciprocal effect possible	Altered absorption of both EGCG and drug	Case reports of interactions	[108]
β-blockers	Propranolol, Metoprolol	Hypertension, heart failure, arrythmias, angina	EGCG inhibits intestinal transport/metabolism	↓ Bioavailability of β-blockers (drug side)	Reciprocal PK effect: EGCG may also increase systemic exposure	[109]

## 8. Strategies to Enhance Bioavailability of Epigallocatechin Gallate

Given EGCG’s inherent instability and low oral bioavailability, multiple strategies have been developed to enhance systemic exposure, as shown in Table 4. Simple interventions such as administering EGCG on an empty stomach can increase absorption two- to fourfold, although fasting administration raises safety concerns related to hepatotoxicity at high doses [110]. Formulation-based approaches have demonstrated greater potential: in both preclinical and clinical investigations, phospholipid complexes (phytosomes) and nanoparticle carriers (liposomes, bilosomes, transferosomes) improve intestinal permeability and shield EGCG from pH- and enzyme-mediated degradation, leading to increased plasma concentrations [111].

The addition of ascorbic acid to formulations stabilizes EGCG by preventing oxidative degradation and simultaneously enhances absorption [112]. Experimental strategies such as pro-drug synthesis (peracetylated pro-EGCG) [113] and co-administration with metabolism inhibitors like piperine have also demonstrated improved pharmacokinetic profiles in preclinical models [114]. All these developments point to the importance of formulation science as a necessary condition for the effective translation of EGCG into clinical treatments.

A critical limitation, however, is that most bioavailability-enhancing strategies remain validated primarily in preclinical models, with limited head-to-head clinical comparisons. Ensuring translational success will require systematic evaluation of safety, scalability, and inter-individual variability, particularly given EGCG’s dose-dependent hepatotoxicity and microbiome-modulated metabolism.

**Table 4:** Strategies to enhance EGCG bioavailability and pharmacokinetics.

Strategy	Mechanism/Rationale	Examples/Approaches	Key Outcomes	Limitations/Considerations	Reference
Nanoencapsulation/Nanoparticles	Protects EGCG from degradation; increases solubility and intestinal absorption	- EGCG-loaded liposomes (phospholipid vesicles) - Polymeric nanoparticles (PLGA, chitosan, PEGylated systems) - Solid lipid nanoparticles (SLN)	↑ Stability in GI tract ↑ Plasma concentration Controlled release	Complexity in formulation; scale-up issues; regulatory hurdles	[115,116,117]
Protein/Peptide Carriers	Binding to proteins improves stability and transport across membranes	- Casein micelles - Gelatin nanoparticles - BSA–EGCG complexes	Sustained release Reduced oxidation Enhanced intestinal uptake	Allergenicity concerns (milk proteins); may alter taste	[118,119,120,121]
Phospholipid Complexes (Phytosomes^®^)	Conjugation with phospholipids enhances lipophilicity and membrane permeability	EGCG–phosphatidylcholine complexes (commercial: Greenselect^®^ Phytosome)	2–4× ↑ oral bioavailability Better tissue distribution	Cost; formulation stability	[122,123]
Co-administration with Bioenhancers	Inhibits efflux transporters and metabolic enzymes (COMT, UGTs)	- Piperine (from black pepper) - Quercetin - Ascorbic acid (vitamin C prevents auto-oxidation)	↑ Systemic exposure Reduced EGCG glucuronidation/sulfation	Possible herb–drug interactions May alter safety profile	[20,124,125]
Prodrug Approaches	Mask polar groups to improve absorption, later hydrolyzed in vivo	- Esterified EGCG derivatives - Peracetylated EGCG	↑ Lipophilicity ↑ Plasma half life	May reduce intrinsic activity; prodrug activation variability	[113]
Encapsulation in Polysaccharides	Protects from pH and enzymatic degradation; controlled release	- EGCG in alginate beads - Cyclodextrin inclusion complexes	Sustained colonic release Improved taste masking	Encapsulation efficiency varies; limited load capacity	[126,127]
Lipid-based Formulations	Enhance solubility and lymphatic transport	- Self-emulsifying drug delivery systems (SEDDS) - Nanoemulsions/microemulsions	↑ Solubility Avoids first-pass metabolism	Stability and scalability challenges	[128]
Metal/Mineral Conjugates	Chelation reduces degradation; modulates transport	EGCG–Zn, EGCG–Se complexes	Enhanced antioxidant and anticancer potency Greater plasma stability	Toxicity risk if not controlled	[129,130]
Targeted Delivery Systems	Directs EGCG to tissues/organs of interest	- Antibody–EGCG conjugates - Folate-decorated nanoparticles (for tumor targeting)	Selective accumulation in tumors or inflamed tissue	High cost; complex validation	[131,132]
Controlled-release Formulations	Sustained release prevents rapid clearance	EGCG hydrogels, matrix tablets	Prolonged half life Steady plasma levels	Patient compliance; release variability	[133]
Microbiota-directed Strategies	Modulate gut microbiota to favor beneficial EGCG metabolites (e.g., valerolactones)	- Prebiotics/probiotics co-administration - Engineered gut bacteria	↑ Production of bioactive metabolites Personalized nutrition approach	Still experimental; inter-individual variability	[134]

## 9. Pharmacokinetics: Half-Life

EGCG demonstrates rapid gastrointestinal absorption with peak plasma concentrations within 1–2.5 h, but its therapeutic utility is limited by a short half-life (1.9–4.6 h), extensive phase-II metabolism (methylation, sulfation, glucuronidation), and <1% urinary excretion of unchanged compound, resulting in low bioavailability despite distribution into tissues, including across the blood–brain barrier. Multiple advanced formulation strategies have been developed to overcome these limitations [20]. Prodrug chemistry, exemplified by per-O-acylated derivatives such as EGCG octaacetate (Pro-EGCG), masks phenolic groups to reduce early conjugation, achieving up to 5-fold higher bioavailability, enhanced stability, and sustained therapeutic effects through enzymatic hydrolysis at target sites. Nanocarrier systems—including PLGA nanoparticles that enhance cellular uptake up to 10-fold, and PEGylated liposomes that yield 3–4 times higher plasma concentrations with selective release in acidic tumor environments, demonstrate encapsulation efficiencies of 51–97% and particle sizes < 300 nm, with ligand modifications enabling targeted delivery. Albumin-binding conjugates, such as fatty acid–EGCG derivatives, exploit FcRn-mediated recycling, with palmitic acid conjugation producing 20-fold stronger albumin binding and 5-fold longer serum half-life, paralleling clinical successes like Evans blue derivatives. Lipid-based self-emulsifying drug delivery systems (SEDDS/SMEDDS) enhance solubilization, lymphatic uptake, and membrane permeability, resulting in 2–3-fold increases in oral bioavailability in rodents. However, recent studies caution that chronic use may disrupt gut microbiota. Synergistic co-formulations using stabilizers (ascorbate, xylitol) and bioenhancers such as piperine, which increase EGCG plasma Cmax and AUC by 1.3-fold through 40% inhibition of intestinal glucuronidation, provide dose-sparing benefits and sustained tissue exposure. Recent advances in hybrid delivery systems, including nanoparticle-in-liposome constructs, prodrug-loaded nanocarriers, and stimuli-responsive carriers (pH-, enzyme-, and temperature-sensitive platforms, as well as cell-penetrating peptides for blood–brain barrier penetration) have shown preclinical promise, though translation is challenged by interindividual metabolic variability, off-target risks, and manufacturing standardization. Collectively, these innovations represent a paradigm shift toward precision delivery systems that simultaneously stabilize EGCG, extend its half-life, and optimize parent–metabolite exposure for diverse clinical applications.

## 10. Molecular Pathways in Heart Failure: Targets for EGCG

### 10.1. Oxidative Stress, Keap1–Nrf2 Antioxidant Defense, and Therapeutic Potential of Epigallocatechin Gallate

Reactive oxygen species (ROS), including superoxide anions, hydroxyl radicals, and hydrogen peroxide, arise from normal cellular metabolism—primarily mitochondrial oxidative phosphorylation and enzymes such as NADPH oxidase, xanthine oxidase, and nitric oxide synthase [135,136,137,138]. While low levels of ROS function as secondary messengers in physiological signaling [139,140], excessive accumulation disrupts redox homeostasis, induces oxidative stress, and damages DNA, lipids, and proteins [135,137,139]. This redox imbalance is particularly harmful in cardiomyocytes due to their high mitochondrial activity and limited regenerative capacity [141]. Notably, EGCG exerts its cardioprotective and anti-cancer effects largely through modulation of ROS generation and scavenging, thereby restoring redox balance and preventing oxidative injury [135,136,137,138,139,140,141].

**Figure 3:**
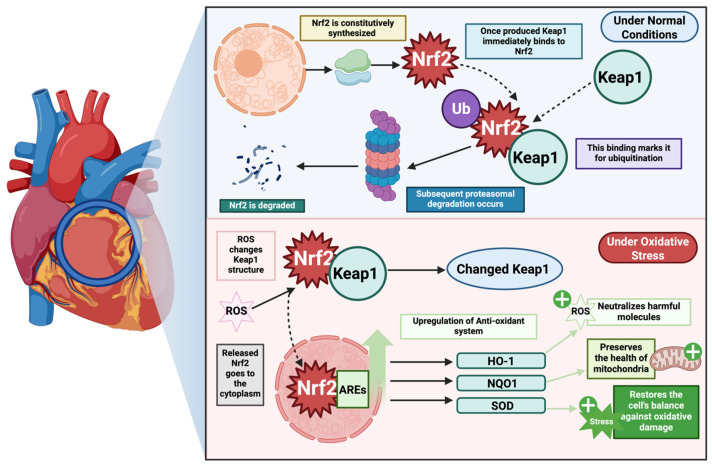
Keap1-Nrf2 axis mediated antioxidant activity. The figure highlights the keap-1-Nrf2 pathway, with a key highlight on Nrf2’s activity in the antioxidant defense. Nrf2 is constitutively synthesized and, under basal conditions, targeted for proteasomal degradation via Keap1. However, under normal conditions when there is no ROS-mediated stressor, Keap1 protein binds to Nrf2 preventing it from conducting the downstream signaling axis. However, in the presence of ROS-mediated stress, Keap1 changes its molecular structure, allowing Nrf2 to act as a transcription factor and upregulate the transcription of key antioxidant defense enzymes—HO-1, NQO1, SOD—allowing them to neutralize the ROS, preserve mitochondrial health, and restore balance. This system is dysregulated in heart failure, allowing ROS-mediated stress to condition, damaging the cell, leading to adverse cardiogenic outcomes.

To maintain redox homeostasis, cells have evolved complex defense mechanisms against oxidative stress, among which the Keap1-Nrf2 pathway plays a critical role [140,142,143]. Nrf2 (nuclear factor 2 related to erythroid factor 2), a transcription factor, acts as a major sensor for oxidative and electrophilic stresses [140,141], while Keap1 (Kelch-like ECH-associated protein-1) protein regulates the function of Nrf-2 [140,142]. Under basal conditions, Nrf2 is sequestered into the cytoplasm by Keap1, which facilitates its ubiquitination and proteasomal degradation [140,142,143]. Hence, under unstressed conditions, Nrf2 is synthesized but constantly degraded, maintaining only a low level [143]. However, under oxidative stress, the cysteine residues in Keap1 become oxidized, leading to conformational changes in Keap1. As a result, Nrf2 dissociates from the complex, stabilizes, and translocates to the nucleus [139,140,142,143]. In the nucleus, it binds to antioxidant response elements (ARE) and upregulates the expression of antioxidant enzyme, such as heme oxygenase-1 (HO-1), NADPH quinone oxidoreductase-1 (NQO1), glutamate–cysteine ligase catalytic and modifier subunits (GCLC, GCLM), glutathione peroxidase (GPx), glutathione S-transferase, superoxide dismutase (SOD), and others. [139,142,143]. Collectively, these enzymes detoxify ROS, replenish glutathione stores, and preserve mitochondrial integrity [144,145]. Figure 3 illustrates the Keap1-Nrf2 pathway, highlighting Nrf2’s key role in the antioxidant system.

Clinical and experimental studies have provided considerable evidence that during heart failure, there is increased oxidative stress in the myocardium and at a systemic level [146,147]. This oxidative stress is a pivotal driver in the pathogenesis and progression of heart failure, with ROS levels acting as possible markers of disease severity [148]. The major cause for increased ROS is mitochondrial dysfunction which is caused by disorganization in substrate metabolism and shift of energy production from mitochondrial fatty acid oxidation to glycolytic pathways leading to intracellular lipid accumulation [149,150]. As a result, there is impaired oxidative phosphorylation, abnormal mitochondrial dynamics, and mitochondrial DNA damage [148,151]. Specifically, ETC complexes I and III are defective leading to decreased ATP production, increased electron loss, and hence excessive ROS production [148]. Chronic increases in ROS lead to a disastrous cycle of mitochondrial DNA damage and functional decline, causing further ROS generation and cellular injury [151,152]. Moreover, ROS directly attenuates contractile function by modifying excitation-contraction coupling proteins, activating hypertrophy signaling kinases, mediating apoptosis, and causing extracellular matrix remodeling [148,152]. These cellular events drive myocardial remodeling, fibrosis, and failure [148,150,152].

Despite being a protective system, the Keap1-Nrf2 axis is often compromised in heart failure [143,153,154]. In murine models of transverse aortic constriction (TAC)-induced pressure overload, Nrf2 initially rises transiently during the adaptive hypertrophy phase but declines as maladaptive remodeling develops, eventually causing chronic heart failure [155]. Mice with a knockout of Nrf2 (Nrf2−/−) show exaggerated pathological hypertrophy, fibrosis, and apoptosis, and accelerated transition to heart failure, whereas overexpression of Nrf2 suppresses ROS and protects cardiomyocytes and cardiac fibroblasts from stress-induced growth [155]. Similar findings are observed in ischemic injury models. Following induction of myocardial infarction, Nrf2−/− mice exhibit maladaptive remodeling, left ventricular dilation, reduced cardiac output, and a rapid progression to heart failure, with mortality rates nearly doubled compared to wild-type controls [153].

Conversely, knockout of cardiomyocyte specific Keap1 and activation of Nrf2 in mice with suprarenal abdominal aortic constriction confers protection against pressure overload-induced fibrosis, cell death, and contractile dysfunction, while still maintaining antioxidant gene expression [156]. These models together underscore Nrf2’s essential role in protecting against oxidative stress-induced remodeling post-injury.

Importantly, human data align with these experimental observations. On transcriptomic analysis of patients with dilated or ischemic cardiomyopathy, the Nrf2/Keap1 ratio was revealed to be elevated, suggesting a compensatory activation; however, the expression of key antioxidant and detoxification genes was consistently reduced [154]. This discrepancy between Nrf2 activation and transcriptional efficacy highlights a functional impairment of the pathway in human heart failure. Thus, impaired Keap1–Nrf2 signaling not only reduces antioxidant defenses, but also promotes a vicious circle of oxidative damage, fibrosis, and cardiac dysfunction.

Given its central role, enhancing Nrf2 activity has emerged as a promising therapeutic strategy. As such, epigallocatechin gallate (EGCG), the most abundant catechin in green tea, has attracted attention for its potent antioxidant and cardioprotective properties [22,157]. Due to its polyphenolic hydroxyl groups, EGCG possesses direct antioxidant activity [157,158,159], enabling free radical scavenging to produce more stable phenolic radicals, inhibition of lipid peroxidation, and enhancement of endogenous antioxidant enzyme activity [160].

More importantly, EGCG indirectly boosts antioxidant capacity by modulating the Keap–Nrf2 axis through powerful activation of Nrf2 [157,161,162,163]. Mechanistically, EGCG is speculated to interact with cysteine residues on Keap1, leading to conformational changes that prevent Keap1 from degrading Nrf2 [163]. This facilitates Nrf2 stabilization, nuclear translocation, and transcription of ARE genes [143]

In murine models of coronary heart disease, addition of EGCG activates the Nrf2/HO-1/NQO1 pathway restoring protein expression of Nrf2, HO-1, and NQO1, thereby reducing cardiac tissue damage [162]. Similarly, EGCG enhances activation of Keap1/P62/Nrf2 signaling pathway in mice models with intracranial hemorrhage, inhibiting inflammation, oxidative stress, and apoptosis [157]. Moreover, in diabetic nephropathy models, EGCG’s cardioprotective effects were abolished in Nrf2 knockout mice, highlighting that its protective actions are Nrf2-dependent [163].

These molecular effects translate into significant cardiovascular benefits. EGCG reduces overload-induced cardiac hypertrophy, fibrosis, and apoptosis, while decreasing risk of ischemia-reperfusion injury [22]. It also improves endothelial function, arterial compliance, and blood pressure regulation [158]. Clinical studies further show that regular consumption of green tea extract lowers LDL cholesterol, improves serum antioxidant capacity, and reduces inflammatory markers in individuals with cardiovascular risk factors [20]. These data establish EGCG not only as a direct antioxidant but also as a potent Nrf2 activator with a promising future as an adjunct in the management of cardiovascular disease and heart failure.

While EGCG has been shown to activate Nrf2 and restore antioxidant capacity across diverse preclinical models, the mechanistic fidelity of this interaction remains incompletely defined. Current evidence is largely inferential, relying on surrogate readouts of downstream ARE-driven gene expression rather than direct structural or biophysical demonstration of EGCG–Keap1 cysteine adduct formation. Moreover, although compensatory dysregulation of Nrf2 target gene transcription in human cardiomyopathy highlights a translational paradox, Nrf2 stabilization does not always equate to functional antioxidant reprogramming. Future work must therefore delineate the kinetics and stoichiometry of EGCG–Keap1 interactions using covalent adductomics, mass spectrometry, and crystallography, and integrate these with redox flux analyses in human cardiomyocytes. Equally critical will be the deployment of clinically relevant, bioavailable formulations in large-animal HF models with longitudinal redox imaging, to establish whether Nrf2 activation by EGCG translates into durable attenuation of oxidative injury in vivo.

### 10.2. Inflammation and Fibrosis

**Figure 4:**
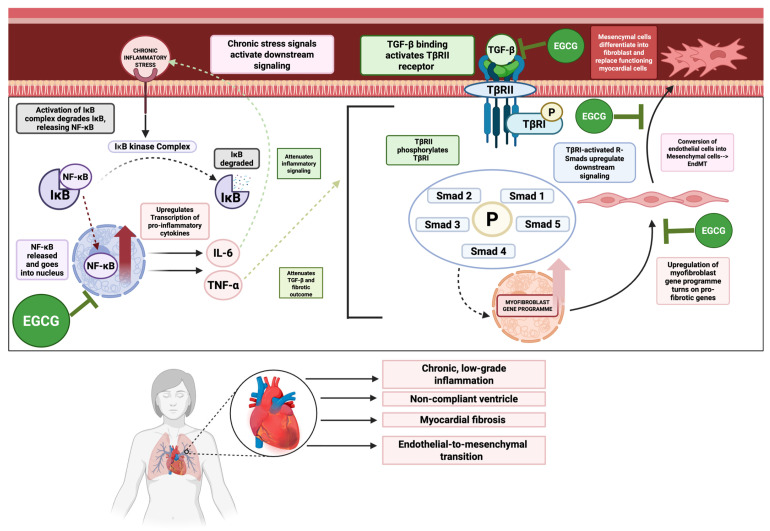
NF-κB, TGF-β, EndoMT mediated inflammation and cardiogenic fibrosis in heart failure. This figure illustrates key mechanisms involved in inflammatory and fibrosis-driven heart failure. NF-κb promotes chronic, low-grade inflammation and its synthesis of TNF-α synergizes with the TGF-β signaling axis to upregulate this axis and its fibrotic outcomes. TGF-β causes downstream signaling and phosphorylation of R-smads, which act as transcription factors to upregulate the myofibroblast gene program. One outcome of this gene program is endothelial-to-mesenchymal transition (EndoMT), in which endothelial factors are switched off, while mesenchymal factors are turned on, allowing these endothelial cells to transform into mesenchymal cells and further mature into fibroblasts that lay down collagen and displace functioning myocytes, thus worsening the fibrogenesis, inflammation, and function in heart failure. However, EGCG can inhibit NF-κB, TGF-β, EndoMT signaling axes, thus attenuating these adverse outcomes in heart failure.

Chronic, low-grade inflammation, often mediated by the NF-κB signaling pathway, contributes significantly to myocardial remodeling and dysfunction in HF (illustrated in Figure 4) [164,165]. This is compounded by progressive myocardial fibrosis, a process largely driven by the TGF-β/Smad signaling axis, which leads to excessive extracellular matrix (ECM) deposition and a stiffened, non-compliant ventricle [166,167,168].

EGCG exerts potent anti-inflammatory effects by inhibiting NF-κB activation, thereby suppressing the production of pro-inflammatory cytokines such as tumor necrosis factor alpha (TNF-α) and interleukin (IL)-6 [159,169,170]. Simultaneously, EGCG modulates the TGF-β1/Smad3 signaling pathway, reducing myocardial fibrosis and ventricular collagen remodeling in mouse models of heart failure [171]. It also effectively inhibits endothelial-to-mesenchymal transition (EndMT), a process where endothelial cells acquire a fibrotic phenotype, which is a key contributor to cardiac fibrosis [172,173].

The preclinical evidence for EGCG’s anti-fibrotic action is robust, targeting both the core signaling pathways and the cellular origin of fibroblasts. However, the lack of human studies evaluating the long-term impact of EGCG on endpoints like left ventricular remodeling and fibrosis, as measured by non-invasive imaging techniques such as cardiac MRI, remains a major translational barrier.

To overcome this translational gap, future work must progress beyond descriptive preclinical models towards precision interrogation of inflammatory–fibrotic circuits. CRISPR/Cas9-based strategies offer a particularly powerful avenue, targeted editing of NF-κB or TGF-β/Smad pathway nodes in human iPSC-derived cardiomyocytes and fibroblasts could definitively establish causal links between EGCG exposure and transcriptional reprogramming. In parallel, CRISPR-mediated fluorescent tagging of Smad3 or NF-κB subunits would permit live-cell tracking of nuclear translocation dynamics under EGCG treatment, providing mechanistic resolution at single-cell level. Equally, genome-wide CRISPR knockout or CRISPRa/i screens could identify previously unrecognized co-factors or enhancers that condition EGCG’s anti-fibrotic efficacy, enabling rational combination strategies. Coupling such molecular precision with non-invasive endpoints such as longitudinal cardiac MRI for fibrosis quantification, and plasma exRNA profiling of fibroblast activation, would yield an integrated translational pipeline, positioning EGCG not merely as a nutraceutical but as a tractable molecular probe for dissecting and modulating inflammatory–fibrotic remodeling in human HF.

### 10.3. Mitochondrial Dysfunction and Energy Metabolism

Heart is an extremely energy-demanding organ, with mitochondria consuming a massive amount of ATP to support continuous contraction. In heart failure, a pathological shift from efficient fatty acid oxidation to a less efficient glucose metabolism, coupled with mitochondrial dysfunction, creates a state of chronic energy starvation [174]. Key regulatory proteins, including AMP-activated protein kinase (AMPK) and peroxisome proliferator-activated receptor gamma coactivator 1-alpha (PGC-1α), govern energy homeostasis and mitochondrial biogenesis [175]. AMPK is expressed in various tissues in the human body, including skeletal muscle, liver, heart, brain, and vasculature. It is a central cellular energy sensor that is activated in states of hypoxia, ischemia, nutritional deprivation (e.g., anorexia and fasting), or exercise that trigger an increased AMP/ATP ratio intracellularly. The main mode of activation of AMPK is through phosphorylation of upstream kinases like LKB1 and Ca^2+^/calmodulin-dependent protein kinase (CaMK). Once activated, AMPK shifts cellular metabolism toward energy-generating catabolic pathways (glucose uptake, glycolysis, fatty acid oxidation) while suppressing ATP-consuming anabolic processes (fatty acid synthesis, cholesterol synthesis, protein synthesis), thereby preserving energy homeostasis [176,177]. It also directly enhances the activity of PGC-1α, a transcriptional coactivator that serves as a master regulator of mitochondrial biogenesis and oxidative metabolism. By partnering with transcription factors such as nuclear respiratory factors (NRF1/2) and estrogen-related receptors (ERRs), PGC-1α drives the expression of mitochondrial transcription factor A (TFAM), which is required for mitochondrial DNA replication and electron transport chain function. Through this network, it increases mitochondrial number and efficiency, promotes fatty acid oxidation, and maintains metabolic flexibility in the heart. In heart failure, PGC-1α expression is reduced, leading to impaired mitochondrial function and energy deficiency [175,178].

**Figure 5:**
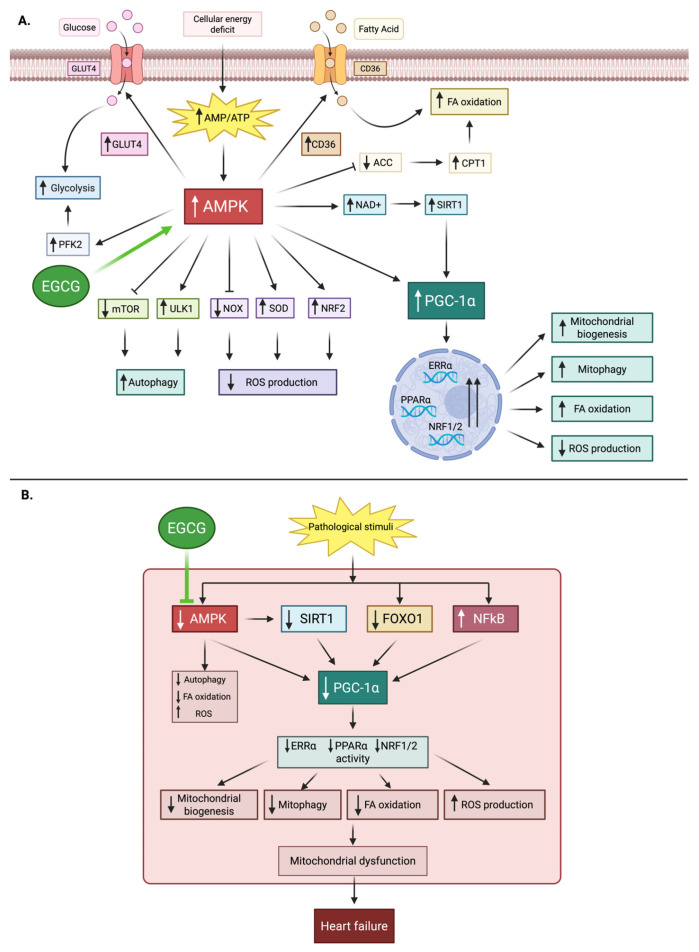
Role of AMPK–PGC-1α signaling in cardiac energy metabolism and the modulatory effects of EGCG under physiological and pathological conditions. (**A**) Physiological conditions: Cellular energy deficit increases AMP/ATP ratio, activating AMPK. This promotes glucose uptake (GLUT4), glycolysis (PFK2), fatty acid (FA) oxidation (via CPT1), autophagy (ULK1, mTOR), antioxidant defense (NRF2, SOD, NOX), and stimulates PGC-1α through SIRT1. PGC-1α enhances mitochondrial biogenesis, mitophagy, FA oxidation, and reduces reactive oxygen species (ROS). EGCG further activates AMPK. (**B**) Pathological conditions: Reduced AMPK activity decreases SIRT1 and PGC-1α signaling, while FOXO1 and NF-κB are dysregulated. This impairs mitochondrial biogenesis, autophagy, FA oxidation, and increases ROS, leading to mitochondrial dysfunction and heart failure. EGCG mitigates these changes by supporting AMPK activation. Abbreviations for Figure 5: AMPK, AMP-activated protein kinase; AMP/ATP, adenosine monophosphate/adenosine triphosphate; EGCG, epigallocatechin gallate; GLUT4, glucose transporter 4; PFK2, phosphofructokinase-2; CD36, cluster of differentiation 36; ACC, acetyl-CoA carboxylase; CPT1, carnitine palmitoyltransferase 1; NAD^+^, nicotinamide adenine dinucleotide; SIRT1, sirtuin 1; PGC-1α, peroxisome proliferator-activated receptor gamma coactivator-1α; PPARα, peroxisome proliferator-activated receptor alpha; ERRα, estrogen-related receptor alpha; NRF1/2, nuclear respiratory factors 1/2; NRF2, nuclear factor erythroid 2–related factor 2; SOD, superoxide dismutase; NOX, NADPH oxidase; ULK1, unc-51-like autophagy activating kinase 1; mTOR, mammalian target of rapamycin; FOXO1, forkhead box O1; NF-κB, nuclear factor kappa-light-chain-enhancer of activated B cells; ROS, reactive oxygen species; FA, fatty acid.

EGCG has been shown to enhance cardiac energy metabolism in pressure overload-induced cardiac dysfunction models [179]. It achieves this by activating the AMPK-PGC-1α axis, which leads to improved fatty acid oxidation, enhanced oxidative phosphorylation, and overall ATP preservation [180,181]. Figure 5 highlights the role of AMPK-PGC-1α in signaling in cardiac energy metabolism and the modulatory effects of EGCG under physiological and pathological conditions. Preclinical studies also demonstrate that EGCG promotes mitochondrial biogenesis and protects against mitochondrial damage in animal models of myocardial injury [182]. The metabolic effects of EGCG are a particularly compelling rationale for its use in HF; however, the data linking EGCG to these specific pathways in human myocardium is non-existent. A crucial next step is to conduct human trials with metabolic endpoints, such as cardiac PET or MRI, to confirm these findings in a clinical setting.

Moreover, to advance beyond preclinical inference, future investigations must deploy cutting-edge mitochondrial phenotyping platforms capable of capturing EGCG’s metabolic effects with cellular and temporal precision. High-resolution respirometry (Seahorse XF and Oroboros O2k systems) coupled with stable isotope-resolved metabolomics (^13^C/^15^N tracers) can delineate flux through fatty acid β-oxidation, glycolysis, and the TCA cycle under EGCG exposure. Super-resolution live-cell imaging modalities such as STED and lattice light-sheet microscopy, combined with mitochondrial membrane potential probes and Ca^2+^ indicators, would permit dynamic visualization of cristae remodeling, fission–fusion kinetics, and organelle-ER crosstalk. Cryo-electron tomography could further resolve ultrastructural alterations in respiratory supercomplexes, while multiplexed immunogold EM would map AMPK–PGC-1α signaling hubs spatially. Integration of these high-content datasets with AI-driven multimodal analytics, graph neural networks trained on imaging, metabolomic, and transcriptomic inputs could yield predictive models of mitochondrial resilience to energetic stress. Ultimately, translation will require validation in human myocardium, leveraging iPSC-derived cardiomyocytes stratified by metabolic genotype, and metabolic imaging endpoints (cardiac PET tracers for fatty acid and glucose uptake, hyperpolarized MRI for real-time fluxes). Such a precision, system-level approach would establish whether EGCG’s mitochondrial effects can be harnessed as a clinically actionable strategy in heart failure.

### 10.4. Apoptosis and Autophagy

The irreversible loss of cardiomyocytes due to apoptosis (programmed cell death) is a hallmark of heart failure, leading to a diminished contractile reserve [183]. Autophagy, a cellular recycling process, is dysregulated in HF; while it can be protective in clearing damaged cellular components, its excessive or maladaptive activation can also lead to cell death [184,185]. EGCG demonstrates a dual regulatory role in these processes. It acts as an anti-apoptotic agent, inhibiting pro-apoptotic caspases and regulating the balance of Bcl-2 and Bax proteins. In rat models of pressure overload-induced cardiac hypertrophy, EGCG was shown to inhibit cardiomyocyte apoptosis and oxidative stress, highlighting its cardioprotective potential [186]. Moreover, in a rat model of myocardial ischemia/reperfusion (I/R) injury, EGCG significantly reduced infarct size and cardiomyocyte apoptosis [187]. Mechanistically, EGCG also modulates autophagy often promoting a protective form of the process to clear damaged mitochondria and cellular debris, thereby improving cell survival [188]. However, the precise regulation of autophagy by EGCG is context-dependent and requires further clarification, particularly in a cardiac setting. More granular studies are needed to differentiate between EGCG’s pro-survival and pro-death effects, especially in different stages of HF.

In line, future studies dissecting EGCG’s dual regulation of apoptosis and autophagy must move beyond rodent hypertrophy and I/R models toward HF-associated CVD contexts with higher translational fidelity, such as diabetic cardiomyopathy, hypertensive heart disease, and chemotherapy-induced cardiotoxicity, each of which embodies oxidative, inflammatory, and metabolic perturbations described in earlier sections. Advanced single-cell multi-omics (scRNA-seq, scATAC-seq, and spatial transcriptomics) applied to failing myocardium could clarify whether EGCG biases autophagy toward a protective, mitophagy-dominant phenotype versus maladaptive self-digestion, and whether this shift is context-specific across disease substrates. High-content imaging platforms, including correlative light–electron microscopy and live-cell FRET biosensors for Bcl-2/Bax dynamics, could directly quantify apoptotic–autophagic crosstalk in real time. Integrating these datasets with artificial intelligence-driven trajectory-inference models, such as pseudotime or graph-learning algorithms, would enable reconstruction of dynamic state transitions across mitochondrial dysfunction, oxidative stress, and fibrosis signatures would establish system-level maps of how EGCG orchestrates survival pathways in cardiomyocytes. Finally, CRISPR-based reporters engineered into iPSC-derived human cardiomyocytes could allow longitudinal tracking of caspase activation and autophagy flux under EGCG, benchmarked against disease-relevant stressors such as hyperglycaemia, pressure overload, or doxorubicin exposure. Such an integrated, disease-stratified pipeline would resolve whether EGCG’s context-dependent effects can be harnessed as a targeted survival modulator in heart failure and its comorbid cardiovascular syndromes.

## 11. EGCG in Myocardial Infarction and Ischemia Reperfusion

A large body of preclinical data from rodent and isolated heart models support EGCG’s protective role in myocardial infarction (MI) and ischemia-reperfusion (I/R) injury [159]. Studies show that EGCG pre-treatment or co-administration can significantly reduce myocardial infarct size, improve left ventricular (LV) developed pressure, and enhance the recovery of LV function [189]. These cardioprotective effects are primarily attributed to its potent antioxidant and anti-apoptotic properties. EGCG has been shown to improve hemodynamic recovery, increase tissue ATP levels, and reduce markers of oxidative and nitrosative stress in isolated perfused rabbit hearts subjected to cardioplegic arrest [190]. These findings present a strong rationale for evaluating EGCG as a potential adjunct therapy to modern reperfusion strategies like percutaneous coronary intervention (PCI). The goal would be to mitigate reperfusion injury, which paradoxically exacerbates myocardial damage after blood flow is restored. Despite the consistent and promising preclinical data, a substantial translational gap exists, with no dedicated human clinical trials evaluating EGCG’s efficacy in patients with acute MI or those undergoing reperfusion therapy. This represents a critical, unmet clinical need and an area of high-priority research.

Hence, a rational next step would be the design of an early phase, multicenter, randomized, placebo-controlled trial evaluating a bioavailable EGCG formulation as an adjunct to percutaneous coronary intervention (PCI) in patients with acute or elective MI. EGCG could be administered via sublingual or lipid-based nanoformulation loading prior to PCI, with continued dosing in the early post-reperfusion period, and outcomes assessed by cardiac MRI endpoints such as infarct size, myocardial salvage index, and microvascular obstruction, together with circulating biomarkers of oxidative/nitrosative stress. Importantly, a factorial design incorporating standard-of-care agents (e.g., high-intensity statins, β-blockers, ACE inhibitors/ARNIs, and P2Y_12_ inhibitors) would allow interrogation of synergistic or additive effects, particularly as statins and renin–angiotensin inhibitors converge mechanistically on redox and inflammatory pathways that EGCG also modulates. Risk-enriched subpopulations such as patients with metabolic comorbidities (type 2 diabetes, obesity, metabolic syndrome) or genetic predisposition to premature MI (e.g., familial hypercholesterolaemia, elevated Lp(a), PCSK9 variants) could be leveraged both as mechanistic models and as trial cohorts where EGCG’s pleiotropic actions may yield amplified benefit. An innovative yet feasible extension would be to integrate AI-enabled multimodal analytics, combining serial metabolic imaging (PET tracers for substrate utilization, hyperpolarized MRI for real-time flux) with plasma exRNA and proteomic signatures, to generate predictive response phenotypes. Such a trial would directly address the translational gap by testing EGCG not merely as a phytochemical antioxidant but as a system-level cardioprotective adjunct within contemporary reperfusion paradigms.

## 12. EGCG and Cardiac Arrhythmogenesis

While less explored than its metabolic and anti-fibrotic effects, EGCG’s potential as an anti-arrhythmic agent is an emerging area of research. It has been shown to modulate the activity of key ion channels that regulate cardiac excitability. EGCG inhibits the cardiac sodium channel Nav1.5 in a dose-dependent manner, primarily by modulating channel inactivation [191]. It also interacts with potassium (K^+^) channels, with some studies showing inhibition of the human ether-a-go-go-related gene (hERG) K^+^ channel, a known target for pro-arrhythmic drugs [192].

**Figure 6:**
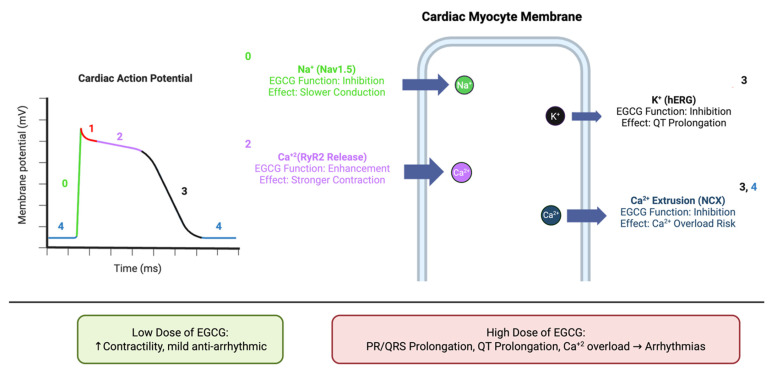
Modulation of cardiac ion channels by EGCG and implications for arrhythmogenesis. Schematic illustration of the cardiac action potential and key ion fluxes modulated by EGCG. Depolarization (Phase 0) is mediated by sodium influx through Nav1.5 channels, repolarization (Phase 3) by potassium efflux via hERG/IKr channels, and calcium handling by RyR2-mediated SR release and NCX extrusion. EGCG inhibits Nav1.5 and hERG currents, enhances RyR2-mediated Ca^2+^ release, and suppresses NCX-mediated Ca^2+^ extrusion. At low concentrations, these effects may increase contractility and confer mild anti-arrhythmic benefit, while at higher concentrations they can prolong PR/QRS and QT intervals, promote Ca^2+^ overload, and predispose to arrhythmias. Note: this schematic highlights only the major ion transporters directly modulated by EGCG; other channels and exchangers that contribute to the cardiac action potential are not depicted.

EGCG’s effects on intracellular calcium (Ca^2+^) handling are particularly notable. At nanomolar concentrations, EGCG increases myocyte contractility by enhancing sarcoplasmic reticulum (SR) Ca^2+^ loading and activating ryanodine receptor channels (RyR2). It also inhibits the sodium–calcium exchanger (NCX), which slows Ca^2+^ extrusion from the cell, further enhancing Ca^2+^ transients and contractility. These effects on Ca^2+^ handling are summarized in Figure 6, which illustrates EGCG’s modulation of Nav1.5, hERG, RyR2, and NCX during the cardiac action potential [193]. The effects of EGCG on cardiac electrophysiology appear to be dose-dependent and biphasic. While low concentrations may provide a positive inotropic effect and stabilize Ca^2+^ handling, high concentrations (as might be found in unregulated supplements) have been shown to prolong PR and QRS intervals and alter the ST-T wave segment, suggesting potential pro-arrhythmic effects [194]. This finding is critical and points to a narrow therapeutic window for EGCG as an anti-arrhythmic agent.

The modulation of cardiac ion channels (e.g., Nav1.5, hERG, RyR2, NCX) is a complex, dose-dependent phenomenon. The ability of EGCG to inhibit Nav1.5 and hERG could theoretically offer an anti-arrhythmic effect (similar to Class I and III anti-arrhythmic drugs), but the simultaneous enhancement of Ca^2+^ transients could also increase the risk of delayed afterdepolarizations and arrhythmias, especially at higher concentrations. Figure 5 further highlights this biphasic nature, contrasting the potential beneficial effects of low-dose EGCG with the pro-arrhythmic risks of high-dose exposure. This intricate relationship means that a simple “more is better” approach with EGCG is not only ineffective due to bioavailability issues but potentially dangerous. Future studies must precisely define the therapeutic window for EGCG’s anti-arrhythmic effects, moving beyond general anti-inflammatory claims to a nuanced understanding of its electrophysiological profile. This also underscores the need for rigorous safety monitoring in any human trials.

Future work must therefore prioritize rigorous delineation of EGCG’s electrophysiological therapeutic window through systematic dose–response studies in cardiomyocytes, engineered heart tissues, and in vivo arrhythmia-prone models, establishing the concentration range that separates beneficial inotropy from pro-arrhythmic liability. Also, advanced in vivo validation should be undertaken in arrhythmia-prone models such as RyR2 or CASQ2 mutant mice for catecholaminergic polymorphic ventricular tachycardia (CPVT), hERG- or KCNQ1/KCNE1-mutant long QT models, coronary artery ligation/reperfusion paradigms, pressure overload (TAC)-induced heart failure, streptozotocin-induced or db/db diabetic cardiomyopathy, spontaneously hypertensive rats, and large-animal post-myocardial infarction canine or porcine systems, to establish translational fidelity of EGCG’s electrophysiological effects. Detailed ion-channel selectivity profiling using patch-clamp and high-throughput electrophysiology across Nav, Kv, hERG, RyR2, NCX, and L-type Ca^2+^ channels is essential to map EGCG’s pharmacodynamic spectrum, while structure–activity relationship approaches could refine the catechin scaffold to retain Nav1.5 inactivation with reduced hERG blockade. Human iPSC-derived cardiomyocytes combined with multielectrode array and optical mapping platforms offer a tractable system for quantifying action potential duration, conduction velocity, and arrhythmic triggers, supported by advanced in vivo validation in models of catecholaminergic polymorphic VT, long QT, and ischemia reperfusion-induced arrhythmias. Given that comorbid conditions such as diabetes, hypertensive heart disease, and heart failure remodel Ca^2+^ handling and ion-channel expression, disease-stratified studies will be critical to contextualize EGCG’s electrophysiological actions. Parallel exploration of combination strategies with established anti-arrhythmics or metabolic modulators (e.g., β-blockers, statins) could reveal synergistic or antagonistic effects mediated through convergent redox and inflammatory axes. Crucially, PK–PD correlation studies linking plasma and tissue concentrations of EGCG (and metabolites) with electrophysiological endpoints will be necessary to establish clinically relevant exposure–response relationships. Finally, translational pipelines should incorporate AI-assisted ECG analytics and machine learning-based arrhythmia prediction to sensitively detect early pro-arrhythmic signatures, thereby enabling the design of first-in-human safety trials with continuous telemetry, QT/QRS interval monitoring, and robust adjudication of arrhythmic events.

## 13. EGCG in Cardio-Oncology

### 13.1. Chemotherapy-Induced Cardiotoxicity

Doxorubicin-Induced Cardiotoxicity (DIC): Doxorubicin is a potent anticancer agent, but its clinical use is limited by a cumulative dose-dependent cardiotoxicity that can lead to heart failure. The mechanism of DIC involves the formation of doxorubicin-iron complexes in mitochondria, leading to an overproduction of ROS, lipid peroxidation, and a specific form of cell death known as ferroptosis [195]. Preclinical studies show that EGCG pre-treatment effectively alleviates DIC by decreasing iron accumulation and inhibiting oxidative stress and abnormal lipid metabolism, the hallmarks of ferroptosis. It also upregulates AMP-activated protein kinase α2 (AMPKα2) and promotes adaptive autophagy to maintain mitochondrial function and energy supply [196,197]. A logical next study would be a controlled preclinical trial in doxorubicin-treated large-animal models, integrating cardiac MRI, echocardiographic strain imaging, and ferroptosis-specific biomarkers such as lipid peroxidation products (MDA, 4-HNE), glutathione depletion, GPX4 downregulation, and iron accumulation indices, to rigorously assess whether EGCG co-administration can attenuate structural, metabolic, and functional hallmarks of DIC in a setting that closely approximates human translation.

Trastuzumab and Tyrosine Kinase Inhibitors (TKIs): Trastuzumab, a HER2-targeted monoclonal antibody, and TKIs are also associated with cardiotoxicity, although through a different, often reversible, mechanism that is not dose-dependent [198]. A key insight is that EGCG can inhibit the activation of key receptor tyrosine kinases (RTKs) such as EGFR, HER2, and HER3, which are crucial for both cancer cell proliferation and cardiomyocyte survival signaling [199,200,201]. By inhibiting these targets, EGCG may protect against the cardiotoxic effects of HER2-targeted therapies. Looking forward, innovative studies could employ CRISPR-based HER2 knock-in cardiomyocyte models, coupled with phosphoproteomic profiling and AI-driven cardiac imaging analytics, to define how EGCG modulates RTK signaling networks under trastuzumab or TKI exposure, thereby establishing a translational framework for cardio-protection in oncology patients.

### 13.2. Shared Pathways with Cancer

**Figure 7:**
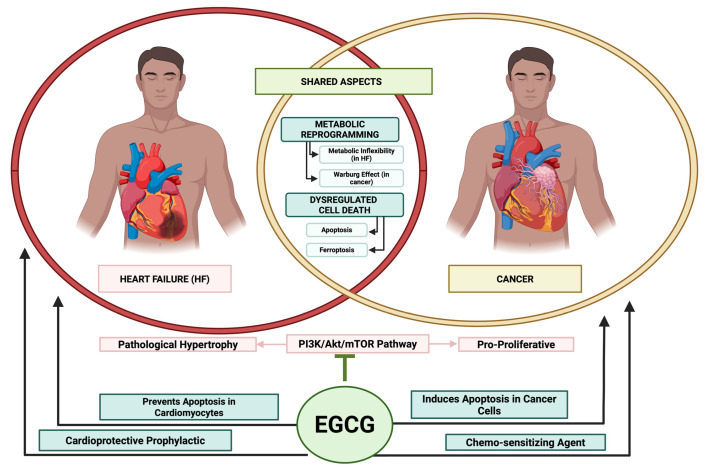
Shared profile in heart failure and cancer and EGCG’s pleiotropic effects in modulating adverse outcomes. This figure illustrates certain shared pathogenic features in heart failure and cancer, such as metabolic programming and dysregulated cell death. However, it also highlights EGCG’s beneficial effects in attenuating the pathogenesis of these conditions. (1) EGCG can inhibit the PI3K/Akt/mTOR pathway that leads to pro-proliferative phase in cancer and pathological hypertrophy in heart failure. (2) EGCG can induce apoptosis in cancer cells to remove pathological cells, while preventing apoptosis in cardiomyositis preserving its functioning. (3) EGCG can also act as agents, specifically chemo-sensitive agents in cancer to improve oncogenic therapeutic efficacy and cardioprotective prophylactic treatment in heart failure to prevent progression in heart failure.

The shared molecular drivers of HF and cancer are a critical theme. Both diseases are characterized by metabolic reprogramming (e.g., the Warburg effect in cancer, metabolic inflexibility in HF), dysregulated cell death (apoptosis, ferroptosis), and altered signaling pathways (e.g., p53, Akt/mTOR) [202,203]. EGCG’s ability to modulate these pathways offers a dual therapeutic advantage. It inhibits the PI3K/Akt/mTOR pathway, which is pro-proliferative in cancer and can lead to pathological hypertrophy in the heart. It also induces apoptosis in cancer cells while simultaneously preventing it in cardiomyocytes [187,204,205]. This suggests that EGCG, particularly in a bioavailable formulation, could serve as a single, multi-target agent for integrated cardio-oncology care, acting as both a cardioprotective prophylactic and a chemo-sensitizing agent. Figure 7 illustrates certain shared pathogenic features in HF and cancer and EGCG’s pleiotropic effects in modulating adverse outcomes.

Cancer and heart failure are complex, heterogeneous diseases driven by multiple, redundant pathways. A single-target drug often leads to the activation of compensatory pathways, reducing its long-term efficacy [206]. EGCG’s ability to modulate numerous signaling nodes—inhibiting RTKs for cancer and protecting against TKI-induced cardiotoxicity, activating Nrf2 for antioxidant defense, and inhibiting NF-κB for anti-inflammation—is a powerful advantage. The same molecule that can mitigate doxorubicin-induced ferroptosis can also inhibit the very RTKs that are overexpressed in cancer cells. This is a revolutionary concept that warrants rigorous clinical investigation.

To translate this dual therapeutic potential, systematic interrogation must begin at the cellular level using human iPSC-derived cardiomyocytes and patient-derived cancer organoids exposed to EGCG, both alone and in combination with conventional chemotherapeutics, to dissect context-dependent effects on apoptosis, ferroptosis, and metabolic reprogramming. These studies should be extended into advanced preclinical platforms such as engineered heart tissue, microfluidic “heart-tumor-on-a-chip” co-culture systems, and large-animal models that recapitulate both oncologic and cardiovascular stressors, thereby capturing the dynamic crosstalk between tumor growth and cardiac adaptation under EGCG treatment. On this foundation, early-phase clinical trials can be designed in high-risk cardio-oncology cohorts, for example breast cancer patients receiving anthracyclines or HER2-targeted therapy, with endpoints including cardiac MRI for functional remodeling, circulating biomarkers of ferroptosis and inflammation, and oncologic response rates. Ultimately, rigorous integration of multi-omics, single-cell transcriptomics, metabolomics, proteomics, and phosphoproteomics, coupled with AI-driven data fusion and predictive modelling, will be essential to resolve EGCG’s pleiotropic signaling effects and identify response signatures. Combining these approaches with CRISPR-based mechanistic validation and adaptive trial designs will provide a system-level framework to determine whether EGCG can indeed function as a unifying agent in cardio-oncology, rather than a compound constrained by context-dependent variability.

### 13.3. PAR-2 Signalling Is a Common Theme in HF and Cancer

Our previous investigations have demonstrated that protease-activated receptor 2 (PAR-2) plays a significant role in inflammation associated with osteoarthritis and cancer. Our results indicate that activation of PAR-2 leads to the stimulation of inflammatory and anti-apoptotic pathways [207,208,209]. PAR-2 is a G-protein-coupled receptor which is activated by serine proteases, such as trypsin and tryptase, which are the majority class of PAR-2 ligands. These act on PAR-2, cleaving between R35–S36, thereby activating it [210,211].

**Figure 8:**
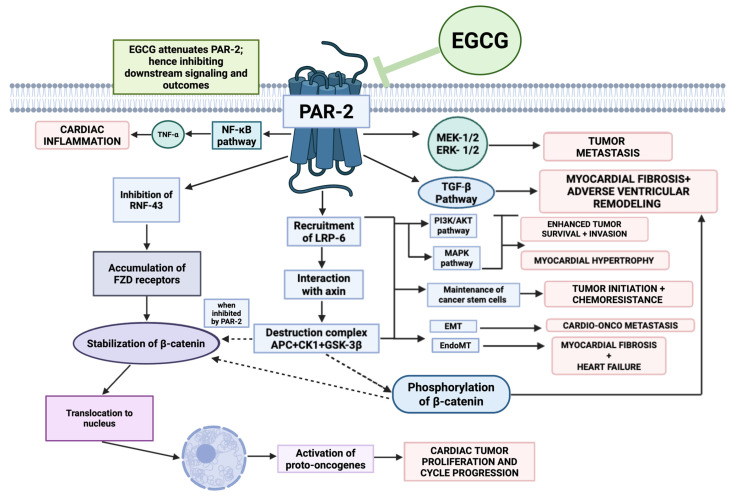
PAR-2 mediated oncogenic and cardiogenic adverse outcomes. PAR-2’s involvement in carcinogenic outcomes has been well established, due to its effects of MEK 1/2, ERK1/2, stabilization of β-cathenin and such pathways leading to pro-proliferative phases, tumor progression, survival, and chemoresistance. However, PAR-2 also has certain involvement in heart failure progression. (1) It stimulates the NF-κB pathway which leads to cardiac inflammation. (2) Via destruction of APC + CK1 + GSK-3β and downstream signaling it also contributes to EndoMT, leading to worsening fibrosis. (3) Phosphorylation of beta-catenin downstream can trigger myocardial fibrosis and ventricular remodeling. (4) PAR-2 mediated MAPK pathway can contribute to myocardial hypertrophy, worsening the prognosis of heart failure. However, as illustrated, certain data have shown the EGCG can downregulate PAR-2, hence attenuate PAR-2 mediated outcomes. Additionally, as illustrated in the figure the presence of bold arrows represents well-established pathways that PAR-2 has been shown to modulate in the literature. The dotted arrows represent a possible mechanism that might be influenced by PAR-2, however further research is required on these signaling to establish them as PAR-2 mediated pathways.

PAR-2 actively participates in both heart failure (HF) and cancer by modulating pathogenic signaling pathways. Figure 8 highlights PAR-2’s involvement in carcinogenic and oncogenic adverse outcomes. In cancer, PAR-2 is highly expressed in colorectal and breast malignancies [212,213], where its activation promotes tumor cell proliferation, migration, drug resistance, and metastatic spread—especially via the β-catenin and NF-κB pathways in colorectal cancer [213,214,215,216,217,218]. In heart failure, PAR-2 signaling contributes to cardiac inflammation and adverse tissue remodeling; experimental studies have linked PAR-2 activation to increased myocardial fibrosis and functional deterioration, exacerbating HF progression [219].

Case in point, Friebel et al. demonstrated in a model of PAR-2 deficient mice with heart failure preserved ejection fraction (HFpEF) that PAR-2 was associated with increased myocardial fibrosis, ECM remodeling, and diastolic dysfunction [220]. Similarly, Antoniak et al. found several heart failure outcomes linked to PAR-2. They found in a model of rat neonatal cardiomyocytes that PAR-2 induced hypertrophic growth, in addition to activating pro-fibrotic chemokines, induced inflammation, and heart failure. However, when studied in a murine myocardial infarction model (induced by blocking of LAD), PAR-2 attenuated heart remodeling and improved cardiac function [219]. Thus, PAR-2 serves as a disease driver in both cancer and heart failure through inflammatory and remodeling mechanisms.

Although our research explored PAR-2 inhibition by EGCG in cancer models, its effects in the context of heart failure require further investigation. We hypothesize that EGCG’s ability to inhibit PAR-2, thereby downregulating NF-κB and related inflammatory pathways, suggests that EGCG could be a potent inhibitor of heart failure and its associated cardio-oncological outcomes.

A decisive next step is a cross-disciplinary program that functionally deconvolves PAR-2 (F2RL1) signaling across tumor–heart axes using matched human systems and dual-disease models. At the cellular level, deploy CRISPR/Cas9 knock-out/knock-in and CRISPRa/i of F2RL1 with biosensor read-outs for Gq/11, G12/13, ERK, NF-κB, and β-arrestin (BRET/FRET) to map biased PAR-2 transduction under EGCG, FXa, trypsin/tryptase, and tumor-derived protease milieus. Couple iPSC-cardiomyocytes/cardiac fibroblasts with patient-derived organoids from CRC (β-catenin/NF-κB–high), HER2^+^ breast cancer (RTK crosstalk), and chondrosarcoma (cartilage ECM/MMP-rich, RANKL axis) in heart–tumor-on-a-chip microphysiology with controllable protease gradients to quantify EGCG ± NOAC (apixaban/rivaroxaban) effects on PAR-2–dependent inflammation, hypertrophy, fibrosis, invasion, and chemo-response. At the tissue scale, apply spatial transcriptomics/proteomics and single-cell multi-omics to hearts and tumors from models with cell-type–specific F2RL1 deletion (cardiomyocyte, fibroblast, myeloid, tumor cell) to resolve context-specific roles, explicitly addressing the MI paradox wherein PAR-2 may be adaptive acutely. In vivo, use the following dual-pathology platforms: (i) CRC/breast/chondrosarcoma xenografts or syngeneic tumors combined with pressure-overload (TAC) or doxorubicin cardiotoxicity; (ii) HFpEF models with implanted tumors to mirror cardio-oncology; and (iii) FXa-high states to test coagulation–PAR-2 coupling. Interventions should compare EGCG (bioavailable formulation) with PAR-2-selective antagonists/pepducins, mast-cell stabilisers, FXa inhibition, and pathway-selective (“biased”) PAR-2 modulators; exploratory modalities could include AAV-delivered dominant-negative PAR-2 in the heart or PROTAC-like degraders for tumour-PAR-2, with stringent safeguards to preserve anti-tumor efficacy. Quantify outcomes by cardiac MRI (fibrosis, strain), PET metabolic imaging, invasive hemodynamics, metastatic burden, and a circulating biomarker panel (PAR-2 cleavage neo-epitopes, tryptase, D-dimer, MMPs, osteolytic mediators). Finally, integrate these data via AI-driven multimodal fusion to derive PAR-2 activity scores and EGCG response signatures, enabling metabotype- and tumor genotype-stratified hypothesis-testing in early human studies (e.g., anthracycline/HER2-therapy cohorts with prospective protease-axis phenotyping), thereby establishing whether EGCG-centered, PAR-2-targeted combinations can deliver true disease modification across HF–cancer continua.

## 14. Translational and Clinical Perspectives

### 14.1. Preclinical Evidence

As summarized in Table 5, a vast body of preclinical evidence supports EGCG’s cardioprotective effects. Studies in various animal models, including pressure overload-induced HF, myocardial infarction, and chemotherapy-induced cardiotoxicity, consistently demonstrate that EGCG administration leads to improved left ventricular function, reduced pathological remodeling, and attenuated cell death [179,221].

### 14.2. Human Data

A major limitation of EGCG research is the paucity of human clinical trials with dedicated cardiac endpoints. While existing trials have evaluated EGCG for a variety of conditions, including metabolic syndrome and hypertension, and have provided encouraging data on vascular health, they do not directly prove its efficacy in preventing or treating heart failure or cardiotoxicity. The lack of randomized controlled trials (RCTs) in these specific populations is a significant and repeatedly noted gap.

Accordingly, a rational program should progress through (i) Phase Ib PK/PD and safety studies of a bioavailable EGCG formulation (e.g., phytosome/SEDDS with acidic stabilizers) in HF phenotypes (HFrEF, HFpEF) and cardio-oncology cohorts (anthracycline and/or HER2-therapy recipients), incorporating fed/fasted arms, hepatic safety gating, and transporter-interaction substudy (OATP1A2/2B1, BCRP, P-gp) to quantify drug–nutrient interactions with β-blockers/ACEi/ARNI; (ii) Phase IIa mechanistic RCTs—enriched by metabolic comorbidity (T2D, obesity, metabolic syndrome) or genetic risk (familial hypercholesterolemia, high Lp(a))—testing EGCG vs. placebo as adjunct to guideline-directed therapy (factorial with SGLT2i/ARNI to probe synergy), with primary endpoints on CMR (infarct scar/ECV, T1/T2 mapping), speckle-tracking GLS, CPET VO_2_peak, and circulating panels (NT-proBNP, hs-troponin, galectin-3, ST2, IL-6/TNF-α, oxylipins); oncology arms should add ferroptosis markers (MDA, 4-HNE, GPX4, GSH/GSSG, labile iron) and serial echo/GLS during chemotherapy. (iii) Phase IIb–III pragmatic trials in high-risk cardio-oncology (e.g., early breast cancer on anthracyclines ± trastuzumab) and HFpEF with inflammatory–metabolic signatures, powered for hard cardiac outcomes (HF hospitalization, ≥10% relative change in GLS/ECV at 6–12 months) with hierarchical testing of quality-of-life (KCCQ) and arrhythmia burden (continuous ambulatory ECG). Designs should be adaptive, with biomarker-guided response enrichment (microbiome “metabotypes”, COMT/UGT genotypes, baseline bile-acid/lipidomic profiles) and exposure–response modelling linking parent EGCG + conjugates to target engagement (e.g., Nrf2 gene set activation scores, NF-κB/TGF-β pathway readouts). Safety oversight must include liver function stopping rules, QT/QRS telemetry (given dose-dependent ion-channel effects), and a pre-specified prohibition/subgroup for bortezomib-treated patients due to known antagonism. Finally, a platform trial architecture spanning HF and cardio-oncology domains can evaluate EGCG alone vs. combinations (e.g., EGCG + statin, EGCG + SGLT2i, EGCG + FXa inhibition in PAR-2-high states), with embedded multi-omics (plasma proteomics, metabolomics, cfRNA) and AI-driven multimodal fusion (imaging + omics + PK) to derive predictive response signatures and refine dosing/regimen selection for phase III.

**Table 5:** Preclinical and clinical studies of EGCG in cancer prevention and therapy.

Study Type	Model/Participants	EGCG Intervention	Observed Effects and Mechanistic Insights	Main Outcomes	Reference
Preclinical (in vitro and in vivo)	Rat cardiac fibroblasts in vitro (Ang II stimulation); Rat pressure-overload hypertrophy via abdominal aortic constriction (AAC) in vivo	EGCG 50 mg/kg (in vivo); EGCG in μM range in vitro (exact dose not stated)	Anti-fibrotic and anti-hypertrophic: EGCG significantly reduced collagen synthesis, fibronectin expression, and proliferation in cardiac fibroblasts under Ang II. In AAC-induced hypertrophy, EGCG ameliorated cardiac fibrosis, blunting connective tissue growth factor (CTGF) overexpression. Mechanistically, EGCG blocked NF-κB activation (less p65 nuclear translocation, preserved IκB-α) and thereby suppressed CTGF induction.	Prevented pathological cardiac remodeling in pressure overload; first evidence that EGCG’s cardioprotection in hypertrophy is via NF-κB/CTGF inhibition.	[222]
Preclinical (in vivo)	Wistar rat myocardial infarction model (isoproterenol-induced MI)	EGCG 10, 20, 30 mg/kg orally, daily for 21 days (pretreatment)	Antioxidant cardioprotection: EGCG dose-dependently lowered myocardial lipid peroxidation and elevated antioxidant enzyme levels in heart tissue. 30 mg/kg had the strongest effect. EGCG maintained the cardiac redox defense system, mitigating ISO-induced oxidative damage. No adverse effects observed. Mechanisms are attributed to free-radical scavenging and antioxidant effects of EGCG.	Reduced MI injury: EGCG prevented oxidative cardiac damage and improved lipid profiles in MI rats, thereby limiting infarct severity and preserving tissue integrity.	[223]
Preclinical (in vivo)	Type 2 diabetic rat model (diabetic cardiomyopathy)	EGCG 40 mg/kg or 80 mg/kg orally, daily for 8 weeks	Anti-fibrotic and cardiometabolic effects: In diabetic hearts, EGCG improved left ventricular contractile function and reduced hypertrophy (lower heart weight index) and injury markers. It significantly alleviated myocardial fibrosis (less collagen I/III deposition, lower hydroxyproline) by downregulating pro-fibrotic TGF-β_1_ and MMP-2/9 levels. Notably, EGCG activated cardiac autophagy—increased LC3 and Beclin1—via AMPK upregulation and mTOR inhibition, which in turn repressed the TGF-β/MMP fibrotic pathway.	Improved cardiac function in diabetic cardiomyopathy: EGCG enhanced LV function and reduced fibrosis in diabetic rats. Mechanistically, benefits were linked to autophagy activation (AMPK/mTOR) and suppression of TGF-β-mediated fibrosis, highlighting a novel cardioprotective pathway.	[224]
Clinical (pilot observational)	Patients with transthyretin amyloid cardiomyopathy (ATTR-CM), wild-type (senile) form (*n* = 25, all males, age ~71)	Green tea extract capsules providing 600 mg EGCG daily, ≥12 months	Cardioprotective (amyloid-stabilizing): After 12 months of EGCG, left-ventricular mass decreased by ~6% (from median 196 g to 180 g, *p* = 0.03) and no significant increase in interventricular wall thickness or decline in ejection fraction occurred (in untreated ATTR-CM, wall thickness typically increases and EF falls). This suggests a halt in disease progression. Total cholesterol also dropped ~8%. Mechanistic basis: EGCG binds and stabilizes TTR, and prior studies show it can disrupt amyloid fibrils, consistent with the observed stabilization/regression of cardiac amyloid. No serious adverse effects reported.	Disease stabilization in cardiac amyloidosis: Chronic EGCG intake halted cardiac hypertrophy progression in ATTR-CM patients, evidenced by stable or reduced LV mass and wall thickness, which implies slowed amyloid deposition. This pilot outcome indicates EGCG’s potential to modify disease course in a human heart failure etiology lacking other disease-modifying therapy.	[225]
Clinical (open-label trial)	Pediatric patients with restrictive or hypertrophic cardiomyopathy (*n* = 12, ages ~1–14) with diastolic heart failure (impaired relaxation)	Decaffeinated green tea catechin extract (Life Extension^®^), titrated up to ~50 mg/kg/day EGCG (325 mg EGCG per capsule) over 3 months, then continued for 12 months	Improved diastolic function: EGCG therapy led to a significant decrease in isovolumetric relaxation time (IVRT, from 115 ms at baseline to 94 ms at 12 months, *p* ≈ 0.03) and an increase in stroke volume and end-diastolic volume (e.g., stroke volume 25→30 mL at 12 mo, *p* ~0.02) indicating better filling capacity. Left atrial sizes tended to decrease and BNP (heart failure biomarker) levels fell in most patients. Systolic function (EF) and wall thickness remained stable (no deterioration). Mechanistically, green tea catechins (especially EGCG) can bind cardiac troponin and reduce myofibrillar Ca^2+^ sensitivity, improving relaxation. Indeed, authors note EGCG acts as a Ca^2+^-desensitizer, downregulating hypercontractile signaling (potentially via SIRT1-p53 or HDAC pathways as seen in animal models). Tolerability was good with no significant side effects.	Symptomatic and functional relief in diastolic HF: 1 year of high-dose EGCG improved cardiac relaxation and output in children with diastolic dysfunction, as reflected by shortened IVRT and increased volumes. Clinically, patients had improved exercise tolerance and no adverse events. This suggests EGCG as a potential adjunct to enhance diastolic function in heart failure with preserved EF, though controlled trials are needed.	[226]
Preclinical (multiple in vitro/in vivo studies)	Various cancer cell lines (e.g., breast, colon, prostate, lung) and tumor-bearing animal models across cancer types	Typical in vitro EGCG ~10–100 µM; In vivo as green tea extracts or pure EGCG at human-equivalent doses (e.g., 0.1–0.5% in diet or ~50–100 mg/kg)	Broad anti-cancer activities: Extensive studies show EGCG exerts anti-proliferative, pro-apoptotic, anti-angiogenic, and anti-metastatic effects in cancer. EGCG can induce cell-cycle arrest (e.g., G_0_/G_1_ arrest via downregulating cyclins/CDKs), trigger apoptosis (intrinsic pathway via mitochondrial depolarization and caspase activation), and inhibit tumor angiogenesis (reduced VEGF production and microvessel density). It modulates multiple oncogenic signals—interfering with EGFR, PI3K/Akt, MAPK, NF-κB, and other pathways—while upregulating tumor suppressors (e.g., p53, PTEN) and DNA repair mechanisms. These pleiotropic actions target hallmarks of cancer, resulting in slower tumor growth and reduced invasiveness in models.	Suppressed tumor growth and spread: In diverse preclinical models, EGCG treatment consistently slows tumor proliferation, promotes cancer cell death, and blocks angiogenesis and metastasis, thereby reducing tumor burden. This multi-targeted efficacy suggests EGCG as a promising adjunct or chemopreventive agent against various cancers.	[227,228]
Preclinical (in vitro)	Human colorectal cancer cell lines (HCT-116 and HT-29)	EGCG 20–50 µM (approximate range used in vitro)	Anti-metastatic mechanism: EGCG was shown to inhibit the hepatocyte growth factor/c-Met signaling pathway in these colon cancer cells. c-Met is a key driver of tumor invasion and metastasis. By blocking Met activation, EGCG reduced downstream pro-migratory signaling (like MAPK and AKT), thereby suppressing cancer cell migration and invasion. Notably, this effect was independent of H_2_O_2_ (i.e., not merely due to antioxidant activity). EGCG also decreased matrix metalloproteinases (MMP-2/9) in similar models (noted in other studies), further hindering metastatic potential.	Reduced metastatic behavior: EGCG attenuated tumor spread in vitro, evidenced by impaired migration/invasion of colon cancer cells. Inhibition of the HGF/c-Met axis by EGCG suggests a specific molecular target through which EGCG can prevent metastasis. This highlights EGCG’s potential to curb metastatic progression in solid tumors.	[229]
Preclinical (in vitro)	Human nasopharyngeal carcinoma (NPC) cell lines (CNE-2 and 5-8F)	EGCG 40 µM (with time-course up to 48–72 h)	Pro-apoptotic and tumor-suppressive: EGCG significantly inhibited proliferation and induced apoptosis in NPC cells. A key finding was EGCG’s effect on epigenetic regulators: it downregulated Sirtuin 1 (SIRT1), an NAD^+^-dependent deacetylase often overexpressed in cancers. SIRT1 reduction led to increased acetylation (activation) of p53, enhancing p53-mediated apoptotic signaling. Consequently, EGCG-treated NPC cells showed higher Bax/Bcl-2 ratio and caspase-3 activation (hallmarks of intrinsic apoptosis). This SIRT1-p53 axis modulation is one mechanism by which EGCG promotes cancer cell apoptosis and growth arrest.	Induced tumor cell apoptosis: EGCG triggered programmed cell death in NPC, correlating with suppression of SIRT1 and reactivation of p53 tumor suppressor function. The result is potent anti-tumor activity in vitro, indicating EGCG can target cancer cell survival pathways (like SIRT1) to overcome growth and survival advantages of tumor cells.	[230]
Clinical (randomized placebo-controlled trial)	Men with high-grade prostatic intraepithelial neoplasia (HGPIN, a premalignant prostate lesion); Italy (*n* = 60)	Green tea catechin (GTC) supplement, 3 capsules daily (total 600 mg GTC/day, ~50–60% EGCG ≈ 300 mg EGCG/day) for 12 months	Chemoprevention of cancer: Only 1 out of 30 men (3.3%) in the GTC-treated group was diagnosed with prostate cancer within 1 year, compared to 9 out of 30 (30%) in the placebo group—a striking ~90% risk reduction. The GTC group also had consistently lower PSA levels (though not statistically significant) and improved LUTS (lower urinary symptom scores) in those with concomitant BPH. Mechanistically, prostate biopsies showed that EGCG-rich catechins lowered expression of proliferation markers (Ki-67) and increased apoptosis in prostatic tissue (per prior reports). No significant side effects were noted; liver function remained normal and adherence was high.	Reduced cancer incidence: Green tea catechin supplementation significantly prevented prostate cancer development in high-risk men. This landmark proof-of-principle showed EGCG-containing catechins can treat premalignant lesions safely and effectively, presumably by anti-proliferative and pro-apoptotic effects on early tumorigenic foci. It positions EGCG as a potential chemopreventive agent for prostate cancer.	[231]
Clinical (Phase II trial, single arm)	Patients with early-stage chronic lymphocytic leukemia (CLL), Rai stage 0–II, asymptomatic (n = 42); USA (Mayo Clinic)	Polyphenon E green tea extract, 2000 mg EGCG per dose, twice daily (4000 mg EGCG/day) for up to 6 months	Anti-leukemic activity: High-dose EGCG was well tolerated; only 3 patients (7%) had grade 3 adverse events (mild transaminitis, abdominal pain, fatigue). Clinically, 31% of patients had a sustained ≥20% decrease in absolute lymphocyte count (peripheral blood leukemia cells), and 69% of those with enlarged lymph nodes achieved ≥50% reduction in lymph node size. Overall, 69% of patients met criteria for a biological response (lymphocyte count and/or lymph node reduction) during treatment. Plasma EGCG levels achieved correlated with magnitude of node shrinkage (R = 0.44). No patients progressed to requiring chemotherapy while on EGCG. Mechanistic studies indicate EGCG induces apoptosis of CLL B-cells and downmodulates B-cell receptor signaling; this trial’s results align with those effects (many patients had tumor cell count reductions).	Leukemia control in vivo: Oral EGCG (Polyphenon E) elicited partial tumor responses in early CLL, evidenced by significant declines in leukemia cell counts and lymphadenopathy in the majority of patients. While not a cure, EGCG stabilized disease in many cases and was very well tolerated. This represents one of the first clinical demonstrations of EGCG’s anti-cancer activity in humans, supporting further research into EGCG as a therapy or adjunct in low-grade malignancies.	[232]
Preclinical (in vivo)	Mouse solid tumor model with chemotherapy—Sarcoma-180 tumor-bearing mice treated with doxorubicin (DOX)	Co-administration of EGCG alongside DOX; (EGCG dose ~50 mg/kg, given with or before DOX; DOX given at cardiotoxic dose)	Dual anti-cancer and cardioprotective effects: EGCG augmented the chemotherapeutic efficacy of DOX while mitigating its cardiotoxicity. Mice receiving EGCG + DOX had significantly smaller tumors than DOX alone, as EGCG enhanced DOX-induced tumor apoptosis and growth inhibition. Simultaneously, EGCG protected the heart: it reduced DOX-induced cardiomyocyte injury, shown by lower serum LDH release and fewer apoptotic heart cells, versus DOX-only controls. Mechanistically, EGCG co-treatment preserved mitochondrial membrane potential (ΔΨm) and upregulated myocardial MnSOD (antioxidant enzyme), which led to reduced ROS generation and prevention of Ca^2+^ overload in cardiac tissue. These actions culminated in less oxidative damage and cell death in the myocardium. Importantly, EGCG did not interfere with DOX’s tumor-killing ability; it actually improved it.	Chemo efficacy boosted; heart damage reduced: The EGCG + DOX combination enhanced anti-tumor outcomes (greater tumor suppression) while providing cardioprotection against DOX. EGCG prevented DOX-induced heart failure signs (arrhythmias, ultrastructural damage) in mice. Overall, co-therapy resulted in preserved cardiac function and fewer DOX toxic effects, without compromising (and even improving) cancer cell kill. This suggests EGCG as a promising cardioprotective adjuvant in oncology, warranting further clinical investigation.	[233]
Preclinical (in vivo and in vitro)	Doxorubicin-induced cardiotoxicity model (rodents and cardiomyocyte cultures)—Cardio-oncology mechanism study	EGCG pretreatment prior to DOX exposure (doses ~50 mg/kg in rodents; 10–20 µM in cardiomyocytes)	Prevention of ferroptosis (iron-dependent cardiac cell death): EGCG pretreatment was found to alleviate DOX-induced cardiotoxicity by upregulating AMPKα2 and activating adaptive autophagy in cardiomyocytes. Activation of AMPK and autophagy by EGCG led to suppression of ferroptosis, a form of cell death characterized by lipid peroxidation due to iron overload. In DOX-treated hearts, EGCG increased levels of glutathione peroxidase and reduced malondialdehyde, indicating less lipid peroxidation. By curbing ferroptosis, EGCG preserved viable myocardium. This is a distinct mechanism beyond classical antioxidant effects: EGCG essentially triggers the heart’s stress adaptation pathways (AMPK autophagy) to defend against DOX toxicity.	Mechanistic cardioprotection: EGCG shielded the heart from chemotherapy damage through metabolic reprogramming—specifically, inducing autophagy and inhibiting ferroptotic cell death in cardiac muscle. This mechanistic insight reinforces that EGCG’s cardioprotection in cancer therapy is multi-faceted (antioxidant, anti-apoptotic, and now anti-ferroptotic). It provides a rationale for using EGCG or analogs to prevent long-term cardiomyopathy in cancer patients receiving anthracyclines (no clinical trials to date have yet confirmed this benefit in patients, but these findings lay the groundwork for future translational research).	[196]

### 14.3. Safety and Dosing

While EGCG is generally considered safe at dietary levels (e.g., from green tea consumption), there is a well-documented risk of hepatotoxicity at very high doses, particularly with concentrated supplements. Experimental studies in mice have demonstrated that higher bolus doses of EGCG are hepatotoxic in a dose-dependent manner, underscoring the potential risks associated with supplement use compared to tea beverages [234]. However, the direct translation of rodent toxicity data to human risk is limited and should be interpreted with caution.

Mechanistic studies indicate that this hepatotoxicity arises from oxidative stress, driven by suppression of antioxidant enzymes and the overwhelming of the Nrf2 rescue pathway [235,236]. Case reports have linked high-dose green tea extract supplements to acute hepatitis: a 63-year-old woman developed reversible severe hepatitis attributed to EGCG [237]; an 81-year-old Italian woman developed toxic acute hepatitis after one month of Epinerve (90% EGCG) use; and a 72-year-old Italian woman developed cholestatic hepatitis and granulomatous cholangitis after three months of Epinerve and Luteinofta use, with normalization of liver enzymes after treatment and withdrawal [238]. This is a critical safety consideration and underscores the danger of unregulated nutraceutical consumption, highlighting the need for pharmaceutical-grade formulations and standardized dosing under medical supervision.

Across clinical trials, supplemental doses of green tea extract (GTE) have varied widely. For example, Oketch-Rabah et al. report human GTE intake amounts ranged from 500 mg to 3000 mg, which corresponds to approximately 250–1800 mg of EGCG daily. The median intake amount was estimated at 720 mg/day (delivering 623 mg of EGCG daily) [110]. Hepatotoxicity has been reported across a broad dose spectrum, with case reports implicating EGCG doses as low as ~140 mg/day and up to ~1000 mg/day. A regulatory review (EFSA, 2018) noted that daily EGCG intake of ≥800 mg (in supplements) can cause liver enzymes (ALT) to rise above normal levels [110,239]. Additionally, Ramachandran et al. conducted a study where they administered EGCG via intraperitoneal and oral route for fourteen days. A trend of dose- and route-dependent hepatotoxicity was noted, with intraperitoneal causing more hepatotoxicity and increased serum lipid profiles [41].

Subsequently, in a 2016 systematic review of randomized controlled trials, Isomura et al. found only mild, transient elevations in liver enzymes. They concluded that “liver-related adverse events are expected to be rare.” Due to the low number of cases, statistical significance could not be established. One severe liver injury case was reported at a high EGCG dose, but causality could not be confidently attributed to EGCG alone [240]. In the 2020 United States Pharmacopeia (USP) review by Oketch-Rabah et al. included observational reports and case studies, emphasizing rare but serious hepatotoxicity events. Expert reviewers judged most cases as at least “probably” related to high-EGCG formulations. As they have stated: “risks of hepatotoxicity due to GTE intake are real, and exposure may lead to liver injury, including serious liver injury.” [110].

An overall interpretation remains consistent, that there is a need to advocate for safety precautions. Since drug- or herb-induced liver injury usually emerges within ~6 months, routine liver-function monitoring during the first 6 months of GTE therapy is advised. Accordingly, USP guidance now instructs manufacturers to warn users not to take GTE without food (to avoid fasting bioavailability) and to consult a clinician if signs of liver injury occur (e.g., abdominal pain, dark urine, jaundice) [110].

## 15. Limitations of Current Evidence

The most significant limitation of current EGCG research is the profound translational disconnect between the vast body of robust preclinical evidence and the limited clinical data. The central issue remains EGCG’s poor oral bioavailability and rapid metabolism, which makes it exceedingly difficult to achieve and sustain therapeutic plasma concentrations in human subjects. Furthermore, a significant degree of heterogeneity exists in the preclinical literature, including variations in EGCG doses, formulations, animal models, and outcome measures, which complicates the synthesis of a clear evidence base. Finally, the lack of standardized regulation for nutraceuticals versus pharmaceuticals creates a challenging environment for both research and clinical application, raising questions of safety, efficacy, and quality control.

## 16. Future Strategies and Research Directions

The future of EGCG research necessitates a strategic shift from a broad, general approach to a targeted, precision-guided one.

Bioavailability: Future research must prioritize the development and testing of advanced delivery systems. This includes nanocarriers (e.g., pH-responsive, ligand-functionalized nanoparticles) and prodrugs to improve EGCG’s stability, target specificity, and systemic exposure.

Combination Therapy: A promising avenue is to explore synergistic combination therapies. EGCG’s metabolic and anti-fibrotic effects are highly complementary to the recent mechanistic discoveries of sodium-glucose co-transporter 2 (SGLT2) inhibitors, which have revolutionized HF treatment. A combination of EGCG with SGLT2i could offer a multi-pronged attack on the shared pathological pathways of HF.

Precision Nutrition: The response to EGCG is likely influenced by genetic polymorphisms in metabolic enzymes like COMT and glutathione S-transferase (GST). Future studies should adopt a precision nutrition approach, using genotype-guided strategies to identify individuals who would most benefit from EGCG supplementation and to optimize dosing based on their metabolic profile.

Integrative Clinical Trials: The urgent need for clinical validation must be addressed. A translational pipeline is proposed, beginning with Phase I trials of bioavailable EGCG formulations with cardiac biomarkers, followed by dedicated Phase II/III randomized controlled trials. These trials should be specifically designed for high-risk populations, such as cancer patients undergoing cardiotoxic chemotherapy, evaluating EGCG as a preventive adjunct.

## 17. Conclusions

This comprehensive review has established that EGCG is far more than a simple nutraceutical; it is a promising, multi-target molecular therapeutic for the prevention and treatment of heart failure and cardio-oncology. The convergence of preclinical evidence across diverse models demonstrates EGCG’s potent ability to modulate key pathological pathways, including oxidative stress, inflammation, mitochondrial dysfunction, and cell death. However, its clinical translation is currently stymied by significant barriers, primarily poor bioavailability and a lack of dedicated human trials with cardiac-specific endpoints.

## Figures and Tables

**Table 2:** Gut microbiota-mediated metabolism of EGCG.

Step/Pathway	Microbial Action/Enzymes	Metabolites Formed	Representative Bacterial Genera	Notes/Biological Relevance	References
Degalloylation	Microbial esterases cleave gallate moiety from EGCG	Epigallocatechin (EGC) + Gallic acid (GA)	*Bifidobacterium*, *Lactobacillus*	Increases EGC absorption; GA has antioxidant and anti-inflammatory activity	[51,52]
Ring Fission (C-ring cleavage)	Microbial reductases/lyases break C-ring	Valerolactones (e.g., 5-(3,4,5-trihydroxyphenyl)-γ-valerolactone)	*Eubacterium*, *Flavonifractor plautii*	Key intermediates in flavanol metabolism; absorbed in colon	[53,54,55]
Dehydroxylation and Decarboxylation	Dehydroxylases, decarboxylases modify B- and A-rings	Phenylpropionic acids, Phenylacetic acids	*Clostridium*, *Eggerthella lenta*	Simplification of structure enhances systemic absorption	[51,56]
Dehydrogenation and Reduction	Oxidoreductases act on valerolactones	Phenyl-γ-valeric acid derivatives	*Bacteroides*, *Ruminococcus*	Circulating metabolites detected in plasma after green tea ingestion	[51,56]
Further Catabolism	Successive β-oxidation, dehydroxylation	Hippuric acid, Benzoic acid derivatives	Mixed microbiota (various Firmicutes, Bacteroidetes)	Excreted in urine; major end-products of flavanol metabolism	[51]

## Data Availability

No new data were created or analyzed in this study.
